# Diversity and biogeographical patterns of legumes (Leguminosae) indigenous to southern Africa

**DOI:** 10.3897/phytokeys.70.9147

**Published:** 2016-10-04

**Authors:** Marike Trytsman, Robert H. Westfall, Philippus J. J. Breytenbach, Frikkie J. Calitz, Abraham E. van Wyk

**Affiliations:** 1 Agricultural Research Council - Animal Production Institute, Lynn East, 0039 South Africa; 2 Department of Plant Science, University of Pretoria, Hatfield, 0028 South Africa; 3 Independent researcher, Garsfontein, 0081 South Africa (deceased); 4 Agricultural Research Council - Central Office (Biometry Services), Hatfield, 0028 South Africa

**Keywords:** Agriculture, agronomy, assemblages, biogeography, biomes, bioregions, breeding, diversity, ecology, Fabaceae, flora, floristics, fodder, growth form, legumes, leguminochoria, Leguminosae, pastures, phytochoria, soil conservation, South Africa, southern Africa, species range, species richness, vegetation

## Abstract

The principal aim of this study was to establish biogeographical patterns in the legume flora of southern Africa so as to facilitate the selection of species with agricultural potential. Plant collection data from the National Herbarium, South Africa, were analysed to establish the diversity and areas covered by legumes (Leguminosae/Fabaceae) indigenous to South Africa, Lesotho and Swaziland. A total of 27,322 records from 1,619 quarter degree grid cells, representing 1,580 species, 122 genera and 24 tribes were included in the analyses. Agglomerative hierarchical clustering was applied to the presence or absence of legume species in quarter degree grid cells, the resultant natural biogeographical regions (choria) being referred to as leguminochoria. The description of the 16 uniquely formed leguminochoria focuses on defining the associated bioregions and biomes, as well as on the key climate and soil properties. Legume species with a high occurrence in a leguminochorion are listed as key species. The dominant growth form of key species, species richness and range within each leguminochorion is discussed. Floristic links between the leguminochoria are established, by examining and comparing key species common to clusters, using a vegetation classification program. Soil pH and mean annual minimum temperature were found to be the main drivers for distinguishing among legume assemblages. This is the first time that distribution data for legumes has been used to identify biogeographical areas covered by leguminochoria on the subcontinent. One potential application of the results of this study is to assist in the selection of legumes for pasture breeding and soil conservation programs, especially in arid and semi-arid environments.

This paper is dedicated to the memory of Robert Howard (Bobby) Westfall (17 December 1944–21 January 2016), vegetation ecologist and friend whose sudden death during the preparation of this manuscript deprived us of an invaluable collaborator.

## Introduction

The legume family (Leguminosae; alternative name Fabaceae) is considered one of the largest, most economically significant plant families (Yahara et al. 2013). It is the third largest angiosperm family with about 19,400 species (Lewis et al. 2005) and its considerable importance in agriculture, its ability to occupy different habitats and diverse life forms are well documented (Yahara et al. 2013). Projects such as the Global Legume Diversity Assessment are a first step in studying the rapid loss of legume species diversity. Asia is proposed to be the first continent to be assessed, resulting in a publication on legume diversity in South East Asia (Raes et al. 2013). As reported by Sprent et al. (2010), the full potential of African indigenous legumes has not yet been realised and South Africa is seen as a valuable source of legumes for possible agricultural use in arid and semi-arid regions. However, Yahara et al. (2013) calculated that currently almost 30% of Leguminosae in South Africa are threatened or are of conservation concern. Greater diversification in the use of legume species for food and forage is also acknowledged as vital in a changing world (Sprent et al. 2011).

Most phytogeographical studies of southern Africa aim to describe plant biogeographical regions (Acocks 1953, Goldblatt 1978, White 1983, Cowling et al. 1998, Linder 2001, Van Wyk and Smith 2001, Bredenkamp et al. 2002, Linder et al. 2005, Steenkamp et al. 2005, Mucina and Rutherford 2006, Linder et al. 2012, Linder 2014). Linder et al. (2012) defined a biogeographical region as a set of grid cells more similar in species composition compared to any other grid cells. It is noteworthy that of all the biogeographical regions in southern Africa, the Cape Floristic Kingdom/Region, confined in its entirety to South Africa, is considered globally the most species-rich temperate flora (Linder 2014) and the only major floristic region matching the range of a single broad vegetation type or biome, in this case the Fynbos biome (Van Wyk and Smith 2001).

For southern Africa, Goldblatt (1978) recognized six floristic units, namely five phytogeographic Regions and one Transition Zone: 1) the Zambezian Region; 2) the Karoo-Namib Region; 3) the Tongaland-Pondoland Region; 4) the Afromontane Region; 5) the Cape Region; and 6) the Kalahari-Highveld Transition Zone. In a recent reassessment of sub-Saharan phytochoria (areas possessing a large number of endemic taxa), the Cape Floristic Region was clearly delineated from the surrounding Namib-Karoo and Eastern Karoo phytochoria (Linder et al. 2005). Local foci of floristic endemism in southern Africa are described by Van Wyk and Smith (2001) but, for our purpose here, only those regions and centres of endemism corresponding to the classification of grid cells clustering as phytochoria based on the presence/absence of species of Leguminosae, henceforth referred to as leguminochoria, will be compared for their floristic attributes and congruence.

The use of herbarium collection data to generate outcomes such as species richness and biogeographical regions poses several potential limitations (Robertson and Barker 2006, De la Estrella et al. 2012). Sampling efforts may not be consistent, with some quarter degree grid cells (QDGCs) sampled excessively owing to geographical bias (along main roads or in a nature reserve), taxonomic bias (species that are easy to collect or more conspicuous) and temporal bias (collected in one season). QDGCs have historically been used in many African countries for mapping biodiversity data (Larsen et al. 2009). Other weaknesses include: 1) incorrect identification of specimens; 2) outdated taxonomy and 3) incorrect geo-referencing (Soberón and Peterson 2004). The first two comply with the so-called ‘Linnean shortfall’ as defined by Hortal et al. (2015). The Leguminosae data obtained from the South African National Herbarium (PRE) Computerised Information System (PRECIS) evidently suffered from the deficiencies as stated above. Furthermore, the mean area of 675 km^2^ for a QDGC is a fairly large area to categorise in terms of bioregions, biomes, and climatic and soil properties. Some QDGCs lie in ecotonal areas and could therefore not be accurately classified. Hufkens et al. (2009) define an ecotone as a multi-dimensional environmentally stochastic interaction zone between ecological systems with characteristics defined in space and time, and by the strength of the interaction. The history of PRECIS is summarised by Gibbs Russell et al. (1989), and Steenkamp et al. (2005) provide additional information. Despite the shortcomings of herbarium records, they often remain the only available source of major significance with regard to relevant distribution data (Amici et al. 2014).

The principal aim of the present study is to examine the biogeographical patterns displayed by the indigenous Leguminosae in southern Africa and to determine how the resultant broad scale floristic units compare with other such units, i.e. to distinguish ecologically interpretable phytochoria. In the present contribution, hierarchical clustering was applied to distinguish discrete groups that can be named and classified (Kreft and Jetz 2010), the resultant natural regions (choria) being referred to as leguminochoria. In addition to its plant geographical significance, information gathered in this study and the wealth of descriptive and distribution data accumulated by botanists and taxonomists will be of considerable value to plant breeders or rangeland scientists in their search for legume species with pasture and or soil conservation potential, e.g. the need to select increased drought, acidic and salinity tolerant legumes is essential in the light of future predictions of water shortages (Graham and Vance 2003, Niang 2014).

## Methods

### Distribution data

The Leguminosae records in the South African National Herbarium (PRE) Computerised Information System (PRECIS) were obtained in 2008 and used to map distribution patterns of all species. The recorded presence/absence of species in QDGCs was used for data analysis. The original database contained 33,726 records. Species present outside South Africa, Lesotho and Swaziland were removed, and duplicate records, invalid botanical names, synonyms as well as alien and naturalized legume species were omitted (Trytsman et al. 2011, Trytsman 2013). The edited data resulted in 27,322 records. Where geographical outliers for individual species were noted (–i.e. where a species was recorded outside its main ecological region) it was assumed that the outlier populations was adapted to the given local environmental conditions, and it was therefore not removed from the dataset.

However, the PRECIS database has some inherent weaknesses, especially errors regarding the allocation of taxa to QDGCs. It is estimated that QDGCs for approximately 15% of records may be incorrect (Biodiversity Information Officer, pers. comm). It is noteworthy that an extended QDGC standard has been proposed (Larsen et al. 2009) for mapping biodiversity data across the African continent and as an instrument for sharing biodiversity data where laws, regulations or other formal considerations prevent or prohibit distribution of coordinate-level information. The edited Leguminosae PRECIS data resulted in discarding 19% of the records mainly due to incomplete taxa (only genera, missing subspecies or varieties) and QDGC references resulting in the 27,322 records used. The database does not reflect all herbarium records from southern Africa, but mainly those housed in the National Herbarium in Pretoria and some of its satellite herbaria, notably the KwaZulu-Natal Herbarium (NH) in Durban and the Compton Herbarium (NBG) in Cape Town. Despite its inherent limitations, results of the present analysis have been considered sufficiently meaningful to justify the use of this database, the only one of its kind for the study area.

Names of legume species and intraspecific taxa were verified using the section on the family Leguminosae in the “Plants of Southern Africa, an online checklist” of the South African National Biodiversity Institute
(SANBI), at http://posa.sanbi.org/searchspp.php as published in March 2011. Germishuizen and Meyer (2003) was used to describe each species in terms of its growth form, life cycle, height and elevation. These attributes could be useful information in selection and breeding programs. Data on the SANBI website were compared with Germishuizen and Meyer (2003) where discrepancies were found. The reinstatement of *Calobota* Eckl. & Zeyh. and the genus *Wiborgiella* Thunb. were implemented for the division of *Lebeckia* Thunb., whereas the reinstatement of *Euchlora* Eckl. & Zeyh., *Leobordea* Delile and *Listia* E. Mey. and the new genus *Ezoloba* B.-E. van Wyk & Boatwr. were recorded for reclassification of *Lotononis* (DC.) Eckl. & Zeyh. (Boatwright et al. 2009, 2011). For the analyses, 1,580 species representing 122 genera and 24 tribes were considered.

The maps that were used to generate data on climate (mean annual rainfall, mean annual minimum and maximum temperatures) and soil (phosphorus and pH) within each QDGC were supplied by the Agricultural Research Council - Institute for Soil, Climate and Water (ARC-ISCW, 2009). The exchangeable sodium percentage (ESP) assigned to each bioregion was sourced from Nell (2010).

### Statistical analysis

A Multivariate Agglomerative Hierarchical Clustering (AHC) was applied to the presence or absence of legume species recorded in the PRECIS database. The input matrix thus contained the 1,580 recorded legume species and the 1,619 QDGCs enclosed within the borders of southern Africa. Some species were recorded only once, but such rare species were not excluded from the data set. The cluster analysis was performed using XLSTAT 2010.6.01 Software (Addinsoft to MS Excel) applying Euclidean distance for dissimilarity and the Ward’s linkage method for agglomeration to establish and describe functional legume clusters (leguminochoria). Ward’s method is often preferred in broad-scale biogeographical analyses (Kreft and Jetz 2010) and has been applied in several recent biodiversity studies, e.g. Akhani et al. (2013), Divíšek et al. (2014) and Li et al. (2015). The Euclidean distance was used by Biondi et al. (2015) and Abbate et al. (2016) and both Ward’s method and Euclidean distance by Boratyński et al. (2013) and Sirisena et al. (2013) in geographical biodiversity studies. The statistical results of the present study are given in the Supplementary material 1 where five main clusters (termed A–E) were noted with a centroid QDGC. Each of the main clusters (A–E) was then examined for meaningful smaller cluster groups with clear geographical boundaries, thus defining ecologically interpretable leguminochoria. Thereafter a discriminant analysis was performed on the leguminochoria (dependent variable) using the same software and mean annual rainfall, mean annual maximum and minimum temperature, soil phosphorus and soil pH (H_2_O) (explanatory variables) to identify the possible drivers for discrimination.

The bioregions map of Rutherford et al. (2006) was used as a base layer for plotting the different leguminochoria using the QDGCs (dots on map) assigned to each unique leguminochorion. ArcView GIS 3.2, ESRI Inc. 2002 was used to create the layers. The description of each QDGC was thus based on regional maps where one QDGC average 675 km^2^ (± 26 × 26 km). The use of small (megaregional) scale maps as well as assigning abiotic (rainfall, temperature, soil phosphorus, soil pH and ESP) values to an area as large as a QDGC, evidently resulted in a less accurate dataset. This happened especially where two or more bioregions or biomes converged in a QDGC (ecotones), resulting in a considerable loss of descriptive data for many QDGCs. The abiotic data were easier to assign, since QDGCs could be described in transitional terms and classed in a zone closest to those presented in this study. Additional climatology and agrohydrology data (Schulze 2007) were used to describe leguminochoria. These include notes on, for example, extreme maximum temperatures, net primary production, altitude, days of heavy frost per year, monthly solar radiation and extreme cold spells per year.

Species richness for each leguminochorion was calculated by firstly removing duplicate species present in a leguminochorion. The total number of species was then divided by the total number of QDGCs contained in each leguminochorion. The deletion of duplicate species, however, resulted in a lower total number of QDGCs per leguminochorion, i.e. QDGCs that contained only duplicate species were removed from the dataset.

The percentage occurrence of a species was calculated by dividing the total count of an individual species in a leguminochorion by the number of QDGCs present, i.e. if Species A occurred in 30 of the 50 QDGCs assigned to a leguminochorion, it would have a 60% occurrence in that leguminochorion. The first 20 species with the highest occurrence in a leguminochorion were selected as key species. These species are not indicator species (–i.e. species whose abundance in a given area is believed to indicate certain environmental or ecological conditions or suitable conditions for a group of other species), but rather, from an agricultural viewpoint, a species with potential as a pasture crop being more widely adapted and with a higher occurrence than a rare species with a narrow adaptation. A species is labelled diagnostic when its occurrence is 70% or higher in a given leguminochorion. See Supplementary material 2 for a complete list of species recorded in each leguminochorion. Species present in one cluster only are also noted.

The PHYTOTAB-PC vegetation classification program package of Westfall (1992) was used to form assemblages using the 20 key species recorded in each of the leguminochoria derived from the AHC analyses. The aim of classification is defined as the orderly arrangement of objects according to their differences and similarities (Gabriel and Talbot 1984) and thus, for this study, to ascertain whether floristic links between leguminochoria existed. The method of classification is based on minimum entropy (Westfall et al. 1997) and aims to obtain a cluster sequence where cluster-groups can be formed based on floristic similarities and sequenced according to floristic similarities, delimit cluster-groups and to obtain a species sequence where the cluster-groups and their relations are emphasised (Panagos 1995). This program allows the user to decide on the number of groups classified where the accepted minimum percentage difference between groups is 33%. During the analysis, it was established that six groups were formed by increasing the percentage difference between groups to 38%. A further increase up to 50% resulted in no change in the number of groups (remained at six groups) and therefore the analysis was done at the 38% difference between groups. The resultant classification efficiency for the six groups was 86%, higher than the 60% considered adequate for classification (Westfall 1992).

## Results and Discussion

### Leguminochoria of southern Africa

**Figure 1.**
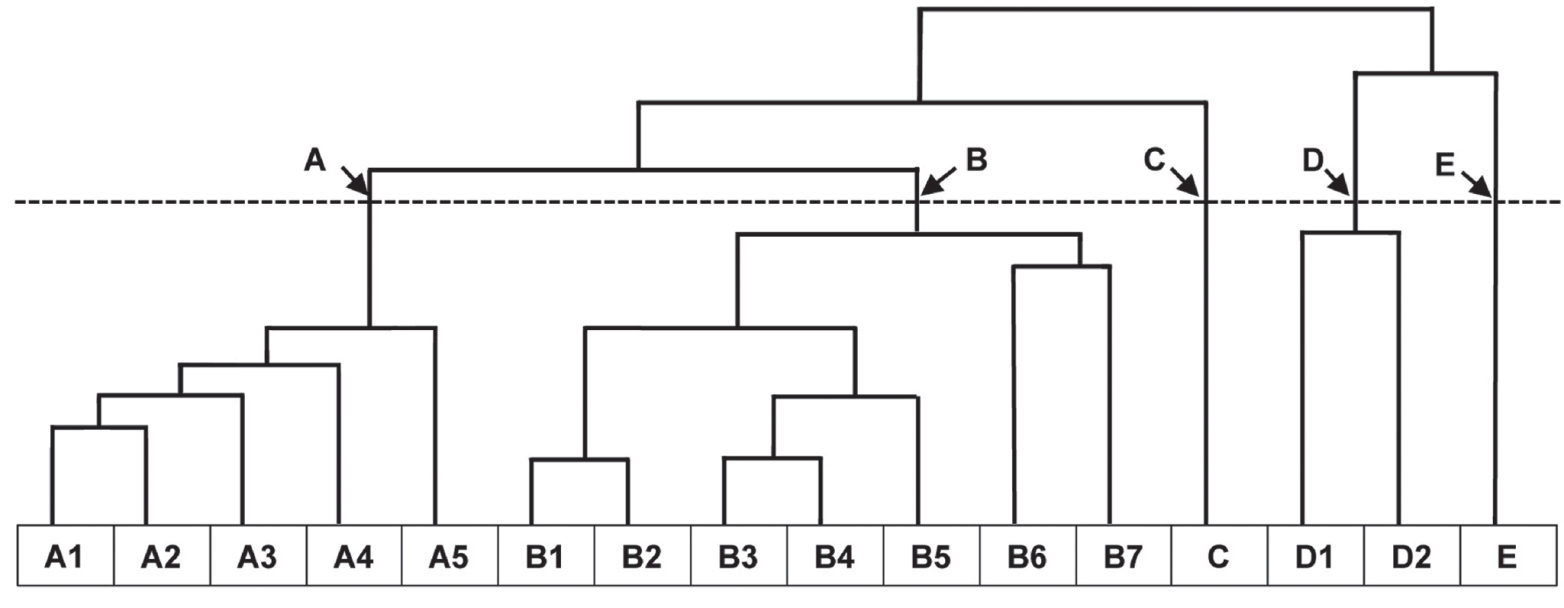
Dendrogram of southern African leguminochoria delimited by Multivariate Agglomerative Hierarchical Clustering. **A1** Southern Afromontane **A2** Albany Centre **A3** Northern Highveld Region **A4** Drakensberg Alpine Centre **A5** Coastal Region **B1** Arid Western Region **B2** Lower-rainfall Cape Floristic Region **B3** Central Arid Region **B4** Generalist Group **B5** Summer Rainfall Region **B6** Northern & Northeastern Savannah Region **B7** Kalahari Bushveld Region **C** Higher-rainfall Cape Floristic Region **D1** Central Bushveld Region **D2** Subtropical Lowveld & Mopane Region **E** Northern Mistbelt.

Figure 1 shows the dendrogram of the five main clusters (A–E) and the subdivisions within each main cluster formed by the clustering analysis. Cluster A, the second largest main cluster, was subdivided into five leguminochoria mainly found in the grassland and savannah regions. Cluster B, the largest main cluster, was subdivided into seven leguminochoria that included one leguminochorion covering a region of South Africa, referred to as the Generalist Group. Cluster C represents the Cape Floristic Region. The two subdivisions of Cluster D represent the savannah regions. Cluster E, the smallest of the five main clusters, represents an Afromontane area. The subdivision of the five main clusters resulted thus in 16 distinct leguminochoria.

The 16 leguminochoria are listed and described in Table 1. The key bioregions (Rutherford et al. 2006) and additional vegetational description (Acocks 1988, Low and Rebelo 1996, Kruger 1999, Van Wyk and Smith 2001, Goldblatt and Manning 2002) delineates the leguminochoria. Leguminochoria B2 and C were formed mainly on the basis of variations in rainfall. Leguminochoria A2 and A4 fall in centres of floristic endemism as described by Van Wyk and Smith (2001). Leguminochorion E is part of the Northern Mistbelt as defined by Mucina and Geldenhuys (2006). Names assigned to the leguminochoria were based on commonly used terms or descriptions contained in the southern Africa vegetation literature.

**Table 1.** Summary of classification of leguminochoria (A1–E) of southern Africa. Key bioregions from Rutherford et al. (2006) with additional descriptions accessed from published literature.

Cluster	Leguminochorion	Key bioregions^1^	Additional description^2^
A	Sourveld and Mixed Veld Group (medium- to high-rainfall areas)		
A1	Southern Afromontane	MHG, SEG, SES	Forest biome (Lo); Moist subtropical (Kr)
A2	Albany Centre	AT, DG, SEG	Albany Centre (Va); Forest biome (Lo); Dry subtropical (Kr)
A3	Northern Highveld Region	CBV, DHG, MHG	Rocky Highveld Grassland (Lo); Moist subtropical (Kr); Bankenveld & N-E Sandy Highveld (Ac)
A4	Drakensberg Alpine Centre	DG, MHG, SEG	Drakensberg Alpine Centre (Va); Forest biome (Lo); Alpine (Kr); *Themeda*-*Festuca* Alpine Veld (Ac)
A5	Coastal Region	IOCB, LV, SES	Maputaland-Pondoland Region (Va); Coastal Bushveld-Grassland (Lo); Moist & humid subtropical (Kr)
B	Seasonal Rainfall Group (all-year, winter and summer rainfall)		
B1	Arid Western Region	NHV, BML	Gariep Centre (Va); Warm desert (Kr); Namaqualand Broken Veld, Succulent Karoo & Strandveld (Ac)
B2	Lower-rainfall Cape Floristic Region	AT, EFR	Maritime (Kr); Coastal Fynbos & Coastal Renosterveld (Ac); Karoo Mountain, Langebaan, Agulhas Plain & Southeastern Centres (Go)
B3	Central Arid Region	EKB, NK	Nama-Karoo and Western Savannah biomes (Ru); Cold & warm desert, Dry subtropical (Kr)
B4	Generalist Group	All regions except: Fynbos, Northern Mistbelt Afromontane, IOCB	Non-specific, Non-Cape group
B5	Summer Rainfall Region	MHG, CBV	
B6	Northern and Northeastern Savannah Region	CBV, LV	Mopane Bushveld, Mixed Lowveld Bushveld, Mixed Bushveld (Lo)
B7	Kalahari Bushveld Region	EKB	Griqualand West Centre (Va); Kimberley Thorn Bushveld & Kalahari Plateau Bushveld (Lo); Kalahari Thornveld (Ac)
**C**	**Higher-rainfall Cape Floristic Region**	EFR, SWF	Mediterranean (Kr); False Sclerophyllous Bush types & Coastal Renosterveld (Ac); mainly Southwestern and Northwestern Centres (Go)
**D**	**Savannah Group**		
D1	Central Bushveld Region	CBV	Moist subtropical (Kr); Springbok Flats Turf Thornveld & Sour Bushveld (Ac)
D2	Subtropical Lowveld & Mopane Region	LV, M	Mopane Bushveld & Mixed Lowveld Bushveld (Lo); Dry and moist tropical (Kr)
**E**	**Northern Mistbelt**	Transitional MHG, LV, CBV	Afromontane Forest (Lo); Inland Moist tropical & moist subtropical (Kr); Tropical Forest Type (Ac)

1 AT : Albany Thicket ; BML : Bushmanland ; CBV : Central Bushveld ; DG : Drakensberg Grassland ; DHG : Dry Highveld Grassland ; EFR : Eastern Fynbos-Renosterveld ; EKB : Eastern Kalahari Bushveld ; IOCB : Indian Ocean Coastal Belt ; LV : Lowveld ; M : Mopane ; MHG : Mesic Highveld Grassland ; NHV : Namaqualand Hardeveld ; NK : Nama-Karoo ; SEG : Sub-Escarpment Grassland ; SES : Sub-Escarpment Savannah ; SWF : Southwest Fynbos .

2 Ac : Acocks 1988 ; Lo : Low and Rebelo 1996 ; Kr : Kruger 1999 , Va : Van Wyk and Smith 2001 ; Go : Goldblatt and Manning 2002 ; Ru : Rutherford et al. 2006 .

### Sourveld and Mixed Veld Group (medium- to high-rainfall areas) (A)

The Sourveld and Mixed Veld Group lies in the medium- to high-rainfall areas of South Africa, Lesotho and Swaziland. This region receives summer rain with frost occurring in the interior. The region is relatively high in net primary production. The Sourveld and Mixed Veld Group is subdivided into five leguminochoria, namely A1: Southern Afromontane, A2: Albany Centre, A3: Northern Highveld Region, A4: Drakensberg Alpine Centre and A5: Coastal Region.

**Figure 2.**
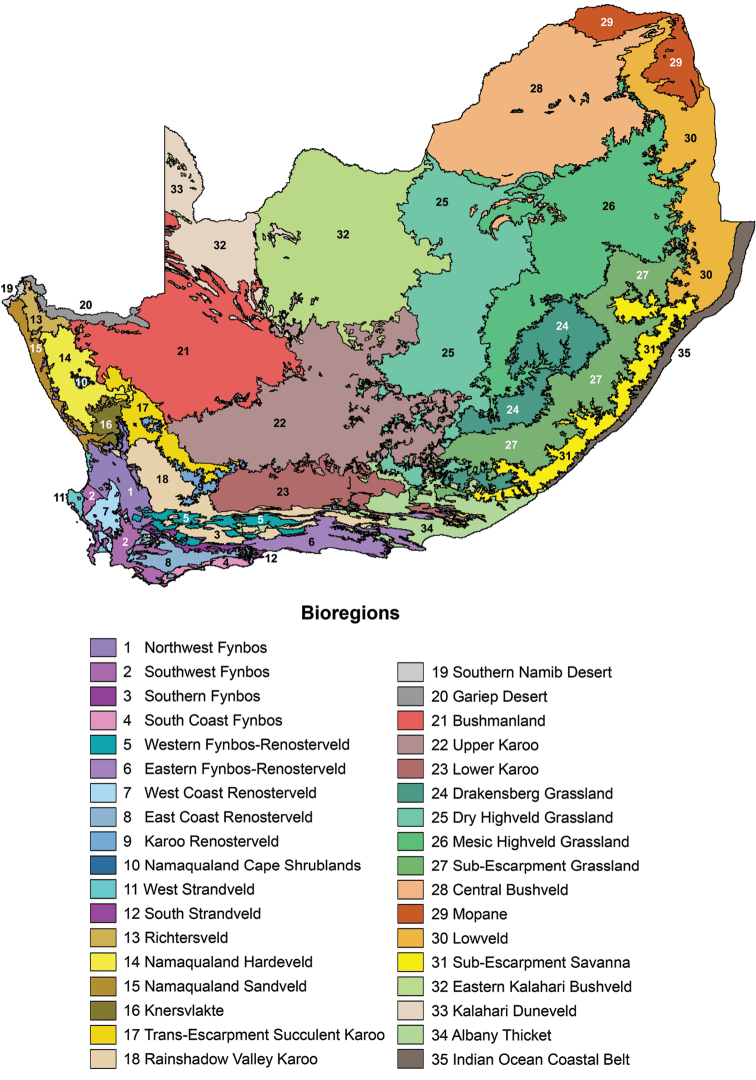
Bioregions of South Africa, Lesotho and Swaziland (Rutherford et al. 2006). The vegetation map shows the 35 bioregions where a bioregion is defined as a composite special terrestrial unit based on similar biotic (vegetation and floristic) and physical features (landscapes and rock types) and processes at the regional scale (Rutherford et al. 2006). The legend should be referred to when comparing the areas covered by leguminochoria.

The 35 bioregions of South Africa, Lesotho and Swaziland as defined by Rutherford et al. (2006) is shown in Figure 2. The legend should be referred to when comparing the areas covered by leguminochoria.

### The Southern Afromontane (A1)

**Figure 3.**
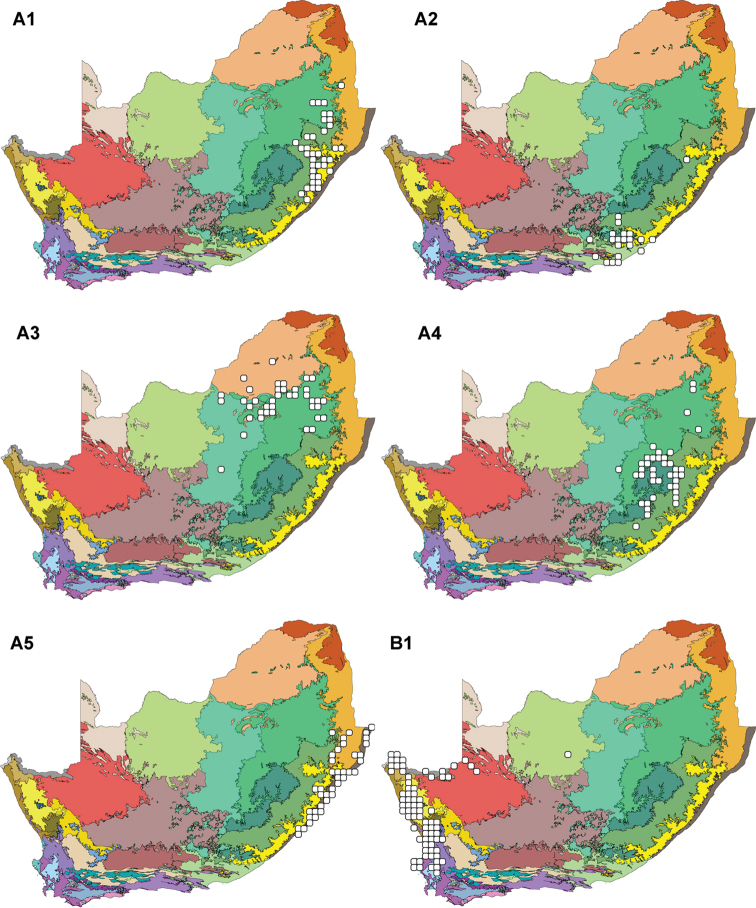
The Leguminochoria **A1–A5** & **B1** superimposed on the Bioregions of southern Africa. Cluster A (Sourveld and Mixed Veld Group) is divided into the Southern Afromontane (**A1**); Albany Centre (**A2**); Northern Highveld Region (**A3**); Drakensberg Alpine Centre (**A4**); and the Coastal Region (**A5**). Cluster B (Seasonal Rainfall Group) is here represented by the Arid Western Region (**B1**); for other subdivisions of cluster B, see Figure 5. The leguminochoria is mapped on bioregions defined by (Rutherford et al. 2006) referring to the legend in Figure 2.

The Southern Afromontane includes legume species mainly confined to the Mesic Highveld Grassland, Sub-Escarpment Grassland and Sub-Escarpment Savannah Bioregions evident from Figure 3 and Table 2. The Grassland biome forms the key biome of this leguminochorion (Table 3). Additional information regarding climatology and agrohydrology (Schulze 2007) is shown in Table 4.

**Table 2.** Representation percentage of key bioregions (Rutherford et al. 2006) within leguminochoria (Cluster A1–E) of southern Africa.

Cluster	A1	A2	A3	A4	A5	B1	B2	B3
AT		**50.0^a^**					**40.0**	
BL						19.1		22.6
CBV			22.2					
DG				35.3				
DHG			16.7					13.0
EFR							**40.0**	
EKB								**26.0**
IOCB					**79.0**			
Low					15.8			
MHG	**50.0**		**61.1**	**41.2**				
NH						**33.2**		
SEG	40.0	**50.0**		23.5				
UK								14.3
**Cluster**	**B4**	**B5**	**B6**	**B7**	**C**	**D1**	**D2**	**E**
CBV	**18.9**	26.6	**40.8**			**100.0**	21.4	22.2
DHG	13.0							
EFR					**61.5**			
EKB	13.1			**95.0**				
Low			**40.8**				**57.2**	
Mop			18.4				21.4	33.3
MHG		**29.8**						**44.5**
SEG		12.9						
SWF					23.1			

a Bold-formatted figures indicate the bioregion with the highest percentage representation in a particular leguminochorion. Only key bioregions with representation values higher than 10% are shown.

AT : Albany Thicket ; BL : Bushmanland ; CBV : Central Bushveld ; DG : Drakensberg Grassland ; DHG : Dry Highveld Grassland ; EFR : Eastern Fynbos Renosterveld ; EKB : Eastern Kalahari Bushveld ; IOCB : Indian Ocean Coastal Belt ; Low : Lowveld ; Mop : Mopane; MHG: Mesic Highveld Grassland ; NH : Namaqualand Hardeveld ; SEG : Sub-Escarpment Grassland ; SWF : Southwest Fynbos ; UK : Upper Karoo .

A1 : Southern Afromontane ; A2 : Albany Centre ; A3 : Northern Highveld Region ; A4 : Drakensberg Alpine Centre ; A5 : Coastal Region ; B1 : Arid Western Region ; B2 : Lower-rainfall Cape Floristic Region ; B3 : Central Arid Region ; B4 : Generalist Group ; B5 : Summer Rainfall Region ; B6 : Northern & Northeastern Savannah Region ; B7 : Kalahari Bushveld Region ; C : Higher-rainfall Cape Floristic Region ; D1 : Central Bushveld Region ; D2 : Subtropical Lowveld & Mopane Region ; E : Northern Mistbelt .

**Table 3.** Representation percentage of key biomes (Rutherford et al. 2006) within leguminochoria (A1–E) of southern Africa.

Leguminochorion	AT	D	FB	GL	IO	NK	SK	SV
A1: Southern Afromontane				**90.9**				9.1
A2: Albany Centre	**50.0** ^a^			**50.0**				
A3: Northern Highveld Region				**81.0**				19.0
A4: Drakensberg Alpine Centre				**100.0**				
A5: Coastal Region					**76.5**			23.5
B1: Arid Western Region		4.6	38.6			6.8	**47.7**	2.3
B2: Lower-rainfall Cape Floristic Region	20.0		**75.0**			5.0		
B3: Central Arid Region	0.6	1.1	1.1	14.8		**38.6**	7.4	36.4
B4: Generalist Group	1.1	1.4	1.7	37.0	0.5	14.5	5.6	**38.2**
B5: Summer Rainfall Region	1.4		0.7	**54.6**	5.0			38.3
B6: Northern & Northeastern Savannah Region								**100.0**
B7: Kalahari Bushveld Region				5.3				**94.7**
C: Higher-rainfall Cape Floristic Region			**100.0**					
D1: Central Bushveld Region								**100.0**
D2: Subtropical Lowveld & Mopane Region								**100.0**
E: Northern Mistbelt				9.1				**90.9**

a Bold-formatted figures indicate the highest percentage biome in a leguminochorion.

AT : Albany Thicket ; D : Desert ; FB : Fynbos ; GL : Grassland ; IO : Indian Ocean Coastal Belt ; NK : Nama-Karoo ; SK : Succulent Karoo ; SV : Savannah .

**Table 4.** Additional information regarding climatology and agrohydrology (Schulze 2007) of leguminochoria (A1–E) in southern Africa. Not all variables are noted with each leguminochorion.

Leguminochorion	Notes on climatology and agrohydrology
A1: Southern Afromontane	36–42°C extreme maximum temperatures, >6 tha^-1^yr^-1^ net primary production, early summer to midsummer rain, 600–1200 mm annual rain, 400–1500 m altitude, <20 days heavy frost/year with frost-free areas
A2: Albany Centre	>40°C extreme maximum temperatures, 2–6 tha^-1^yr^-1^ net primary production, all-year and late and very late summer rain, 200–600 mm annual rain, 0–800 m altitude, <20 days heavy frost/year with frost-free areas
A3: Northern Highveld Region	30–36°C extreme maximum temperatures, 4–8 tha^-1^yr^-1^ net primary production, early summer to midsummer rain, 400–1000 mm annual rain, 800–2000 m altitude, <60 days heavy frost/year, higher monthly solar radiation compared to A1 and A2
A4: Drakensberg Alpine Centre	Mainly <36°C extreme maximum temperatures, 4–10 tha^-1^yr^-1^ net primary production, mainly early summer to midsummer rain, 400–1000 mm annual rain, mainly >2000 m altitude, <80 days heavy frost/year, partly high relative relief, >6 extreme cold spells/year lower than -2.5°C on 3 or more consecutive days, high mountains
A5: Coastal Region	Mainly >40°C extreme maximum temperatures, >4 tha^-1^yr^-1^ net primary production, early to mid- to late summer rain, 600–1200 m annual rain, <800 m altitude, frost-free areas, low to medium relief, mainly sourveld, tropically wet with dry winter season
B1: Arid Western Region	Mainly >44°C extreme maximum temperatures, mainly <2 tha^-1^yr^-1^ net primary production, mainly winter rainfall, <400 mm annual rain, <800 m altitude, mainly frost-free areas and <20 days of heavy frost/year, mainly 25–150 relative relief, high solar radiation during Nov–Feb, sweetveld, arid, hot and dry areas
B2: Lower-rainfall Cape Floristic Region	36–42°C extreme maximum temperatures, 0.5–4.0 tha^-1^yr^-1^ net primary production, all-year rainfall, mainly 200–600 mm annual rain, mainly 0–200 m altitude, mainly frost-free and <40 days heavy frost/year, mainly >50 relative relief, mainly semi-arid, cool and dry
B3: Central Arid Region	<4 tha^-1^yr^-1^ net primary production, mainly late to very late summer rain, mainly between 400–1250 m altitude, mainly <50 relative relief, semi-arid to arid, hot, cool and dry, largely sweetveld
B4: Generalist Group	Extremely diverse in terms of given variables
B5: Summer Rainfall Region	>4 tha^-1^yr^-1^ net primary production, early to mid- to late summer rain, >400 mm annual rain
B6: Northern & Northeastern Savannah Region	Mainly >40°C extreme maximum temperature, midsummer rain, frost-free areas and <20 days of heavy frost, <50 relative relief, sweetveld, semi-arid, hot and dry, the only leguminochorion with 16 occurrences of heat waves >30°C on 3 or more consecutive days/year
B7: Kalahari Bushveld Region	2–6 tha^-1^yr^-1^ net primary production, mainly late summer rain, 200–600 mm annual rain, 1000–1500 m altitude, mainly 20–60 days heavy frost/year, <50 relative relief, sweetveld, semi-arid and dry, plains and pans
C: Higher-rainfall Cape Floristic Region	Mainly 2–4 tha^-1^yr^-1^ net primary production, all-year and winter rain, 400–1200 mm annual rain, frost-free areas, mixed veld, mainly long, dry summers hot or cool
D1: Central Bushveld Region	Mainly 36–40°C extreme maximum temperature, 2–6 tha^-1^yr^-1^ net primary production, early summer to midsummer rain, mainly 400–600 mm annual rain, 600–1500 m altitude, <40 days heavy frost/year, 25–200 relative relief, dry and hot or cool
D2: Subtropical Lowveld & Mopane Region	>40°C extreme maximum temperature, 2–8 tha^-1^yr^-1^ net primary production, midsummer rain, 200–800 mm annual rain, <800 m altitude, mainly frost-free, <50 relative relief, mainly sweetveld, dry and hot
E: Northern Mistbelt	30–40°C maximum extreme temperature, >4 tha^-1^yr^-1^ net primary production, mainly early summer rain, >600 mm annual rain, 600–2000 m altitude, mainly frost-free areas, >50 relative relief, sourveld, long winters, low mountains

**Figure 4.**
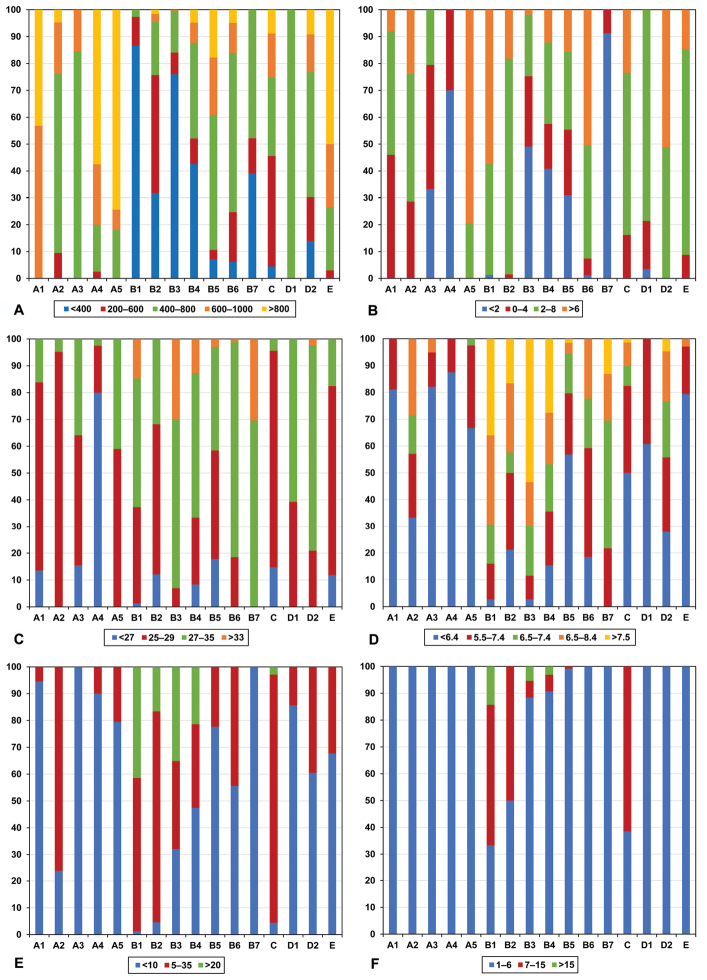
The predominant climate and soil conditions associated with leguminochoria (A1–E) of southern Africa. Climatic conditions shown are mean annual rainfall (**A**) (mm), minimum (**B**) and maximum temperatures (**C**) (°C). The soil properties shown are pH (H_2_O) level (**D**), phosphorus content (mgkg^-1^) (**E**) and exchangeable sodium (**F**) (%). The leguminochoria are termed **A1** Southern Afromontane **A2** Albany Centre **A3** Northern Highveld Region **A4** Drakensberg Alpine Centre **A5** Coastal Region **B1** Arid Western Region **B2** Lower-rainfall Cape Floristic Region **B3** Central Arid Region **B4** Generalist Group **B5** Summer Rainfall Region **B6** Northern & Northeastern Savannah Region **B7** Kalahari Bushveld Region **C** Higher-rainfall Cape Floristic Region **D1** Central Bushveld Region **D2** Subtropical Lowveld & Mopane Region **E** Northern Mistbelt.

A summary of the predominant climate and soil characteristics of these regions is given in Figure 4. Data used to construct Figure 4 is available in Supplementary material 3 (rainfall and temperature) and Supplementary material 4 (soil properties). The high rainfall (>600 mm) and moderate minimum (0–8°C) and maximum (25–29°C) temperatures denote this leguminochorion as a relatively highly productive region. Extreme maximum temperatures of 36–42°C are noted for this leguminochorion (Table 4). Species are adapted to soil with low pH (<6.4), low phosphorus content (<10 mgkg^-1^) and to non-sodic soils.

The Southern Afromontane has some key species in common with the Northern Highveld Region, the Coastal Region, the Summer Rainfall Region and the Northern Mistbelt (e.g. Rhynchosia totta var. totta and Vigna vexillata var. vexillata) (Table 5). High occurrences of different species of *Eriosema* is also noted. A numerical study by Linder et al. (2005) could not retrieve the Afromontane, but here it is clearly defined as the Southern Afromontane (A1) and the Northern Mistbelt (E), with various species related to both leguminochoria. Goldblatt (1978) also noted the presence of mutual key species between the Southern Afromontane and the Coastal Region (e.g. *Crotalaria globifera*, *Dalbergia obovata* and Tephrosia macropoda var. macropoda in this study). This leguminochorion is included in the Maputaland-Pondoland Region (Van Wyk and Smith 2001), Natal (Linder et al. 2005) and Core Afromontane (Steenkamp et al. 2005).

**Table 5.** List of key species recorded in leguminochoria of southern Africa, the occurrence percentage within each leguminochorion (% Occ). Key species preceded by a bullet (•) are present in the designated leguminochorion as key species only and bold-formatted diagnostic species has an occurrence of 70% or higher.

Key species	% Occ
**A1: Southern Afromontane**	
*Argyrolobium tomentosum* (Andrews) Druce	45
• Alysicarpus rugosus (Willd.) DC. subsp. perennirufus J.Léonard	28
• *Argyrolobium speciosum* Eckl. & Zeyh.	39
*Crotalaria globifera* E.Mey.	47
*Dalbergia obovata* E.Mey.	33
*Eriosema cordatum* E.Mey.	69
• *Eriosema distinctum* N.E.Br.	42
*Eriosema kraussianum* Meisn.	58
*Eriosema salignum* E.Mey.	69
Indigofera hilaris Eckl. & Zeyh. var. hilaris	28
• *Leobordea foliosa* (Bolus) B.-E van Wyk & Boatwr.	31
• Lotus discolor E.Mey. subsp. discolor	31
*Otholobium polystictum* (Benth. ex Harv.) C.H.Stirt.	33
• *Pomaria sandersonii* (Harv.) B.B.Simpson & G.P.Lewis	31
• *Rhynchosia cooperi* (Harv. ex Baker f.) Burtt Davy	28
• *Rhynchosia sordida* (E.Mey.) Schinz	28
Rhynchosia totta (Thunb.) DC. var. totta	33
Tephrosia macropoda (E.Mey.) Harv. var. macropoda	33
Trifolium africanum Ser. var. africanum	33
Vigna vexillata (L.) A.Rich. var. vexillata	56
Zornia capensis Pers. subsp. capensis	56
**A2: Albany Centre**	
*Argyrolobium tomentosum* (Andrews) Druce	44
• *Aspalathus chortophila* Eckl. & Zeyh.	40
Aspalathus spinosa L. subsp. spinosa	55
• Calpurnia aurea (Aiton) Benth. subsp. aurea	45
• *Crotalaria obscura* DC.	40
• *Eriosema squarrosum* (Thunb.) Walp.	50
*Indigofera hedyantha* Eckl. & Zeyh.	45
*Indigofera sessilifolia* DC.	40
*Indigofera zeyheri* Spreng. ex Eckl. & Zeyh.	65
• *Lessertia brachystachya* DC.	40
*Melolobium candicans* (E.Mey.) Eckl. & Zeyh.	50
• *Otholobium caffrum* (Eckl. & Zeyh.) C.H.Stirt.	40
*Psoralea oligophylla* Eckl. & Zeyh.	40
*Rhynchosia adenodes* Eckl. & Zeyh.	55
*Rhynchosia caribaea* (Jacq.) DC.	40
• *Rhynchosia ciliata* (Thunb.) Schinz	45
Rhynchosia totta (Thunb.) DC. var. totta	50
• *Schotia latifolia* Jacq.	50
Tephrosia capensis (Jacq.) Pers. var. capensis	65
Trifolium burchellianum Ser. subsp. burchellianum	55
**A3: Northern Highveld Region**	
*Elephantorrhiza elephantina* (Burch.) Skeels	42
• Eriosema burkei Benth. ex Harv. var. burkei	37
*Eriosema cordatum* E.Mey.	34
*Eriosema salignum* E.Mey.	34
• *Erythrina zeyheri* Harv.	34
*Indigofera hedyantha* Eckl. & Zeyh.	34
Indigofera hilaris Eckl. & Zeyh. var. hilaris	47
• *Indigofera oxytropis* Benth. ex Harv.	37
• *Leobordea divaricata* Eckl. & Zeyh.	45
*Leobordea eriantha* (Benth.) B.-E van Wyk & Boatwr.	39
• Pearsonia cajanifolia (Harv.) Polhill subsp. cajanifolia	34
• Pearsonia sessilifolia (Harv.) Dummer subsp. sessilifolia	37
Rhynchosia nervosa Benth. ex Harv. var. nervosa	37
Rhynchosia totta (Thunb.) DC. var. totta	47
• Tephrosia elongata E.Mey. var. elongata	37
Tephrosia longipes Meisn. subsp. longipes var. longipes	47
Trifolium africanum Ser. var. africanum	37
Vigna vexillata (L.) A.Rich. var. vexillata	39
*Zornia linearis* E.Mey.	39
*Zornia milneana* Mohlenbr.	37
**A4: Drakensberg Alpine Centre**	
• *Argyrolobium harveyanum* Oliv.	33
• *Argyrolobium lotoides* Harv.	50
• Argyrolobium rupestre (E.Mey.) Walp. subsp. rupestre	53
• *Argyrolobium tuberosum* (Andrews) Druce	39
• *Dichilus strictus* E.Mey.	42
• *Dolichos angustifolius* Eckl. & Zeyh.	33
*Eriosema salignum* E.Mey.	39
*Indigofera hedyantha* Eckl. & Zeyh.	42
*Leobordea eriantha* (Benth.) B.-E van Wyk & Boatwr.	33
• **Lessertia perennans (Jacq.) DC. var. perennans**	**72**
• *Lotononis galpinii* Dummer	42
*Lotononis laxa* Eckl. & Zeyh.	56
• *Lotononis lotononoides* (Scott-Elliot) B.-E.van Wyk	44
• *Lotononis sericophylla* Benth.	58
*Melolobium microphyllum* (L.f.) Eckl. & Zeyh.	39
• *Melolobium obcordatum* Harv.	42
*Otholobium polystictum* (Benth. ex Harv.) C.H.Stirt.	47
Rhynchosia totta (Thunb.) DC. var. totta	44
Trifolium africanum Ser. var. africanum	44
Trifolium burchellianum Ser. subsp. burchellianum	58
**A5: Coastal Region**	
• *Abrus laevigatus* E.Mey.	51
*Acacia karroo* Hayne	67
• *Aeschynomene micrantha* DC.	54
• Albizia adianthifolia (Schumach.) W.Wight var. adianthifolia	49
***Chamaecrista mimosoides* (L.) Greene**	**82**
• *Crotalaria capensis* Jacq.	62
*Crotalaria globifera* E.Mey.	64
• Crotalaria lanceolata E.Mey. subsp. lanceolata	49
• *Dalbergia armata* E.Mey.	51
*Dalbergia obovata* E.Mey.	67
• *Desmodium dregeanum* Benth.	56
*Eriosema cordatum* E.Mey.	59
• Eriosema parviflorum E.Mey. subsp. parviflorum	64
***Eriosema salignum* E.Mey.**	**77**
• *Neonotonia wightii* (Wight. ex Arn.) J.A.Lackey	49
*Rhynchosia caribaea* (Jacq.) DC.	49
• *Tephrosia grandiflora* (Aiton) Pers.	49
Tephrosia macropoda (E.Mey.) Harv. var. macropoda	49
• Vigna unguiculata (L.) Walp. subsp. unguiculata var. unguiculata	51
Vigna vexillata (L.) A.Rich. var. vexillata	67
**Zornia capensis Pers. subsp. capensis**	**87**
**B1: Arid Western Region**	
Aspalathus acuminata Lam. subsp. acuminata	15
• *Adenolobus garipensis* (E.Mey.) Torre & Hillc.	15
• Aspalathus quinquefolia L. subsp. virgata (Thunb.) R.Dahlgren	15
• Aspalathus spinescens Thunb. subsp. lepida (E.Mey.) R.Dahlgren	22
• *Calobota angustifolia* (E.Mey.) Boatwr. & B.-E.van Wyk	43
• *Calobota sericea* (Thunb.) Boatwr. & B.-E.van Wyk	43
• *Calobota spinescens* (Harv.) Boatwr. & B.-E.van Wyk	19
• *Crotalaria effusa* E.Mey.	20
• Crotalaria excisa (Thunb.) Baker f. subsp. excisa	18
• *Indigastrum argyroides* (E.Mey.) Schrire	23
• *Indigofera amoena* Aiton	16
• *Indigofera exigua* Eckl. & Zeyh.	15
*Indigofera heterophylla* Thunb.	19
• *Indigofera pungens* E.Mey.	16
*Leobordea platycarpa* (Viv.) B.-E van Wyk & Boatwr.	22
• *Lessertia diffusa* R.Br.	28
• *Lessertia excisa* DC.	15
*Lotononis falcata* (E.Mey.) Benth.	27
• *Lotononis parviflora* (P.J.Bergius) D.Dietr.	19
• *Lotononis rabenaviana* Dinter & Harms	15
• *Melolobium aethiopicum* (L.) Druce	20
• *Melolobium humile* Eckl. & Zeyh.	22
*Sutherlandia frutescens* (L.) R.Br.	30
• Wiborgia fusca Thunb. subsp. fusca	15
• *Wiborgia monoptera* E.Mey.	20
• *Wiborgia obcordata* (P.J.Bergius) Thunb.	26
**B2: Lower-rainfall Cape Floristic Region**	
*Acacia karroo* Hayne	22
• Aspalathus collina Eckl. & Zeyh. subsp. collina	31
• Aspalathus hirta E.Mey. subsp. hirta	17
• *Aspalathus hystrix* L.f.	23
• *Aspalathus kougaensis* (Garab. ex R.Dahlgren) R.Dahlgren	18
*Aspalathus nigra* L.	25
• Aspalathus pinguis Thunb. subsp. pinguis	20
• *Aspalathus rubens* Thunb.	32
• *Aspalathus setacea* Eckl. & Zeyh.	26
• Aspalathus shawii L.Bolus subsp. shawii	18
Aspalathus spinosa L. subsp. spinosa	17
• *Aspalathus steudeliana* Brongn.	18
• *Aspalathus subtingens* Eckl. & Zeyh.	31
• *Hypocalyptus sophoroides* (P.J.Bergius) Baill.	17
• *Indigofera denudata* L.f.	17
*Indigofera heterophylla* Thunb.	23
*Lotononis pungens* Eckl. & Zeyh.	28
• *Podalyria burchellii* DC.	20
*Psoralea affinis* Eckl. & Zeyh.	23
*Psoralea oligophylla* Eckl. & Zeyh.	17
• Schotia afra (L.) Thunb. var. afra	22
*Sutherlandia frutescens* (L.) R.Br.	31
Tephrosia capensis (Jacq.) Pers. var. capensis	18
**B3: Central Arid Region**	
*Acacia erioloba* E.Mey.	6
• *Acacia haematoxylon* Willd.	11
*Acacia karroo* Hayne	11
*Cullen tomentosum* (Thunb.) J.W.Grimes	11
*Indigastrum argyraeum* (Eckl. & Zeyh.) Schrire	8
Indigofera alternans DC. var. alternans	29
Indigofera daleoides Benth. ex Harv. var. daleoides	7
• *Indigofera meyeriana* Eckl. & Zeyh.	5
*Indigofera sessilifolia* DC.	10
*Leobordea platycarpa* (Viv.) B.-E van Wyk & Boatwr.	15
• *Lessertia annularis* Burch.	14
• Lessertia macrostachya DC. var. macrostachya	5
• Lessertia pauciflora Harv. var. pauciflora	13
*Lotononis pungens* Eckl. & Zeyh.	5
*Melolobium candicans* (E.Mey.) Eckl. & Zeyh.	24
*Melolobium canescens* Benth.	6
*Melolobium microphyllum* (L.f.) Eckl. & Zeyh.	6
• *Requienia sphaerosperma* DC.	7
Senna italica Mill. subsp. arachoides (Burch.) Lock	12
*Sutherlandia frutescens* (L.) R.Br.	25
• *Sutherlandia humilis* E.Phillips & R.A.Dyer	6
• *Sutherlandia microphylla* Burch. ex DC.	7
**B4: Generalist Group**	
*Acacia karroo* Hayne	8
Crotalaria sphaerocarpa Perr. ex DC. subsp. sphaerocarpa	4
*Elephantorrhiza elephantina* (Burch.) Skeels	3
*Indigastrum argyraeum* (Eckl. & Zeyh.) Schrire	3
Indigofera alternans DC. var. alternans	3
*Indigofera heterotricha* DC.	3
• *Lessertia depressa* Harv.	4
• *Lotononis divaricata* (Eckl. & Zeyh.) Benth.	4
*Lotononis falcata* (E.Mey.) Benth.	3
*Lotononis laxa* Eckl. & Zeyh.	4
• *Lotononis pulchella* (E.Mey.) B.-E.van Wyk	3
• *Melolobium calycinum* Benth.	3
*Melolobium candicans* (E.Mey.) Eckl. & Zeyh.	4
*Melolobium canescens* Benth.	3
*Melolobium microphyllum* (L.f.) Eckl. & Zeyh.	6
• *Parkinsonia africana* Sond.	3
*Rhynchosia adenodes* Eckl. & Zeyh.	3
*Rhynchosia caribaea* (Jacq.) DC.	3
Senna italica Mill. subsp. arachoides (Burch.) Lock	3
*Sutherlandia frutescens* (L.) R.Br.	4
Tephrosia capensis (Jacq.) Pers. var. capensis	4
Trifolium burchellianum Ser. subsp. burchellianum	4
**B5: Summer Rainfall Region**	
*Acacia karroo* Hayne	11
*Chamaecrista mimosoides* (L.) Greene	9
*Elephantorrhiza elephantina* (Burch.) Skeels	8
*Eriosema cordatum* E.Mey.	9
*Eriosema kraussianum* Meisn.	9
*Eriosema salignum* E.Mey.	20
Indigofera hilaris Eckl. & Zeyh. var. hilaris	8
*Indigofera zeyheri* Spreng. ex Eckl. & Zeyh.	7
*Listia heterophylla* E. Mey	7
Mundulea sericea (Willd.) A.Chev. subsp. sericea	16
*Rhynchosia adenodes* Eckl. & Zeyh.	11
Rhynchosia nervosa Benth. ex Harv. var. nervosa	8
Rhynchosia totta (Thunb.) DC. var. totta	30
*Stylosanthes fruticosa* (Retz.) Alston	9
Tephrosia capensis (Jacq.) Pers. var. capensis	8
Tephrosia longipes Meisn. subsp. longipes var. longipes	10
Tephrosia purpurea (L.) Pers. subsp. leptostachya (DC.) Brummitt var. leptostachya	7
• *Tephrosia semiglabra* Sond.	7
Trifolium africanum Ser. var. africanum	20
Vigna vexillata (L.) A.Rich. var. vexillata	9
Zornia capensis Pers. subsp. capensis	17
**B6: Northern and Northeastern Savannah Region**	
*Acacia burkei* Benth.	21
*Acacia caffra* (Thunb.) Willd.	20
Acacia gerrardii Benth. subsp. gerrardii var. gerrardii	19
*Acacia karroo* Hayne	21
*Acacia nigrescens* Oliv.	20
Acacia nilotica (L.) Willd. ex Delile subsp. kraussiana (Benth.) Brenan	19
Acacia tortilis (Forssk.) Hayne subsp. heteracantha (Burch.) Brenan	20
• *Colophospermum mopane* (J.Kirk ex Benth.) J.Kirk ex J.Léonard	18
• Crotalaria monteiroi Taub. ex Baker f. var. monteiroi	18
Dichrostachys cinerea (L.) Wight & Arn. subsp. africana Brenan & Brummitt var. africana	35
• *Faidherbia albida* (Delile) A.Chev.	19
• Indigastrum costatum (Guill. & Perr.) Schrire subsp. macrum (E.Mey.) Schrire	18
Mundulea sericea (Willd.) A.Chev. subsp. sericea	21
*Ormocarpum trichocarpum* (Taub.) Engl.	26
*Peltophorum africanum* Sond.	35
*Philenoptera violacea* (Klotzsch) Schrire	18
• Pterocarpus rotundifolius (Sond.) Druce subsp. rotundifolius	21
Rhynchosia minima (L.) DC. var. minima	18
*Schotia brachypetala* Sond.	20
Senna italica Mill. subsp. arachoides (Burch.) Lock	25
Tephrosia purpurea (L.) Pers. subsp. leptostachya (DC.) Brummitt var. leptostachya	28
• *Xanthocercis zambesiaca* (Baker) Dumaz-le-Grand	19
**B7: Kalahari Bushveld region**	
*Acacia erioloba* E.Mey.	52
• Acacia hebeclada DC. subsp. hebeclada	57
*Acacia karroo* Hayne	39
Acacia tortilis (Forssk.) Hayne subsp. heteracantha (Burch.) Brenan	30
• *Chamaecrista biensis* (Steyaert) Lock	52
• *Crotalaria griquensis* L.Bolus	35
*Crotalaria lotoides* Benth.	30
Crotalaria sphaerocarpa Perr. ex DC. subsp. sphaerocarpa	48
*Cullen tomentosum* (Thunb.) J.W.Grimes	39
*Elephantorrhiza elephantina* (Burch.) Skeels	43
*Indigastrum argyraeum* (Eckl. & Zeyh.) Schrire	30
Indigofera alternans DC. var. alternans	61
• Indigofera cryptantha Benth. ex Harv. var. cryptantha	30
**Indigofera daleoides Benth. ex Harv. var. daleoides**	**83**
*Indigofera filipes* Benth. ex Harv.	61
*Indigofera heterotricha* DC.	43
• Indigofera rhytidocarpa Benth. ex Harv. subsp. rhytidocarpa	30
*Indigofera sessilifolia* DC.	57
*Listia heterophylla* E. Mey	43
• *Rhynchosia confusa* Burtt Davy	61
**Senna italica Mill. subsp. arachoides (Burch.) Lock**	**70**
• ***Tephrosia burchellii* Burtt Davy**	**74**
• *Tephrosia lupinifolia* DC.	30
*Zornia milneana* Mohlenbr.	35
**C: Higher-rainfall Cape Floristic Region**	
Aspalathus acuminata Lam. subsp. acuminata	41
• Aspalathus angustifolia (Lam.) R.Dahlgren subsp. angustifolia	44
• *Aspalathus ciliaris* L.	67
• Aspalathus divaricata Thunb. subsp. divaricata	52
• Aspalathus hispida Thunb. subsp. hispida	58
• Aspalathus juniperina Thunb. subsp. juniperina	33
*Aspalathus nigra* L.	55
• *Aspalathus spicata* Thunb.	45
Aspalathus spinosa L. subsp. spinosa	50
• *Dipogon lignosus* (L.) Verdc.	41
*Indigofera heterophylla* Thunb.	42
• *Lessertia herbacea* (L.) Druce	33
• *Otholobium fruticans* (L.) C.H.Stirt.	41
• *Otholobium polyphyllum* (Eckl. & Zeyh.) C.H.Stirt.	38
• *Otholobium virgatum* (Burm.f.) C.H.Stirt.	35
• *Podalyria myrtillifolia* (Retz.) Willd.	55
*Psoralea affinis* Eckl. & Zeyh.	41
• *Psoralea aphylla* L.	33
• Rafnia capensis (L.) Schinz subsp. capensis	42
• *Rhynchosia capensis* (Burm.f.) Schinz	39
*Sutherlandia frutescens* (L.) R.Br.	45
**D1: Central Bushveld Region**	
*Acacia caffra* (Thunb.) Willd.	68
***Acacia karroo* Hayne**	**82**
• Acacia robusta Burch. subsp. robusta	68
• ***Burkea africana* Hook.**	**79**
*Chamaecrista mimosoides* (L.) Greene	61
*Crotalaria lotoides* Benth.	61
Crotalaria sphaerocarpa Perr. ex DC. subsp. sphaerocarpa	68
Dichrostachys cinerea (L.) Wight & Arn. subsp. africana Brenan & Brummitt var. africana	61
*Eriosema psoraleoides* (Lam.) G.Don	68
*Indigofera filipes* Benth. ex Harv.	64
*Indigofera heterotricha* DC.	64
• *Indigofera melanadenia* Benth. ex Harv.	64
*Listia heterophylla* E. Mey	64
**Mundulea sericea (Willd.) A.Chev. subsp. sericea**	**82**
*Peltophorum africanum* Sond.	61
• Rhynchosia minima (L.) DC. var. prostrata (Harv.) Meikle	64
**Rhynchosia totta (Thunb.) DC. var. totta**	**75**
• ***Sphenostylis angustifolia* Sond.**	**75**
*Stylosanthes fruticosa* (Retz.) Alston	61
**Tephrosia longipes Meisn. subsp. longipes var. longipes**	**79**
*Zornia linearis* E.Mey.	64
**D2: Subtropical Lowveld & Mopane Region**	
*Acacia burkei* Benth.	41
Acacia gerrardii Benth. subsp. gerrardii var. gerrardii	49
*Acacia nigrescens* Oliv.	56
Acacia nilotica (L.) Willd. ex Delile subsp. kraussiana (Benth.) Brenan	54
• Acacia senegal (L.) Willd. var. rostrata Brenan	46
Acacia tortilis (Forssk.) Hayne subsp. heteracantha (Burch.) Brenan	41
• *Albizia anthelmintica* (A.Rich.) Brongn.	49
• Crotalaria laburnifolia L. subsp. australis (Baker f.) Polhill	41
Dichrostachys cinerea (L.) Wight & Arn. subsp. africana Brenan & Brummitt var. africana	66
*Eriosema psoraleoides* (Lam.) G.Don	44
Mundulea sericea (Willd.) A.Chev. subsp. sericea	59
*Ormocarpum trichocarpum* (Taub.) Engl.	61
*Peltophorum africanum* Sond.	61
*Philenoptera violacea* (Klotzsch) Schrire	54
Rhynchosia minima (L.) DC. var. minima	49
Rhynchosia totta (Thunb.) DC. var. totta	49
*Schotia brachypetala* Sond.	56
Senna italica Mill. subsp. arachoides (Burch.) Lock	51
*Stylosanthes fruticosa* (Retz.) Alston	56
Tephrosia longipes Meisn. subsp. longipes var. longipes	44
Tephrosia purpurea (L.) Pers. subsp. leptostachya (DC.) Brummitt var. leptostachya	44
**E: Northern Mistbelt**	
*Acacia caffra* (Thunb.) Willd.	65
• ***Acacia ataxacantha* DC.**	**88**
• *Acacia davyi* N.E.Br.	65
***Acacia karroo* Hayne**	**71**
• Aeschynomene rehmannii Schinz var. leptobotrya (Harms ex Baker f.) J.B.Gillett	65
***Argyrolobium tomentosum* (Andrews) Druce**	**74**
• ***Bauhinia galpinii* N.E.Br.**	**79**
• *Desmodium repandum* (Vahl) DC.	68
***Eriosema psoraleoides* (Lam.) G.Don**	**76**
• ***Indigofera sanguinea* N.E.Br.**	**79**
• *Indigofera tristoides* N.E.Br.	65
• **Pearsonia sessilifolia (Harv.) Dummer subsp. marginata (Schinz) Polhill**	**71**
• **Pseudarthria hookeri Wight & Arn. var. hookeri**	**88**
• *Psoralea arborea* Sims	65
• ***Pterocarpus angolensis* DC.**	**71**
*Rhynchosia caribaea* (Jacq.) DC.	68
• ***Rhynchosia monophylla* Schltr.**	**76**
Rhynchosia totta (Thunb.) DC. var. totta	68
**Vigna vexillata (L.) A.Rich. var. vexillata**	**74**
**Zornia capensis Pers. subsp. capensis**	**82**

### Albany Centre (A2)

In terms of bioregions, the Albany Centre is shared equally in the Albany Thicket and Sub-Escarpment Grassland (Figure 3 and Table 2) and in the Albany Thicket and Grassland biomes (Table 3).

The climate characteristics that prevail in this region (Figure 4) are a medium annual rainfall (400–800 mm), minimum temperatures of mainly 2–8°C and moderate maximum temperatures of 25–29°C. A wide range of soil pH levels is present in this leguminochorion with a phosphorus content of 5–35 mgkg^-1^ and non-sodic soils (Figure 4). The relatively high extreme temperatures (>40°C) noted for this leguminochorion (Table 4) is also noted for the Coastal Region (A5).

The Albany Centre has some key species in common with the Drakensberg Alpine Centre, the Summer Rainfall Region and the Northern Mistbelt (Table 5) (e.g. Rhynchosia totta var. totta) with high occurrences of *Indigofera zeyheri* and Tephrosia capensis var. capensis. Van Wyk and Smith (2001) confirm that floristic elements of many other regions converge in the Albany Centre, although it is not strongly evident in the present study. This leguminochorion forms part of the Kalahari-Highveld Transition Zone and Afromontane (Goldblatt 1978), the Albany Centre (Van Wyk and Smith 2001), Natal (Linder et al. 2005) and the Southern Succulent Karoo and Southeastern Fynbos (Steenkamp et al. 2005).

### Northern Highveld Region (A3)

The Northern Highveld Region does not fall exclusively in the Afromontane; most QDGCs lie within areas of higher altitude and lower rainfall compared to the Southern Afromontane. The Mesic Highveld Grassland is the key bioregion present in this leguminochorion; while Grassland is the biome that is best represented (Figure 3, Table 2 and 3).

The main difference between the Northern Highveld Region (A3) and Southern Afromontane (A1) is the overall lower rainfall (400–800 mm) noted for the former (Figure 4). The low minimum temperatures (mainly <4°C) and the relatively high number of frost days per year (Table 4) occurring in the Northern Highveld Region are also in contrast to the Southern Afromontane. Low pH (<6.4) and low soil phosphorus values (<10 mgkg^-1^) as well as non-sodic soils are noted for this leguminochorion (Figure 4). Schulze (2007) shows that high altitudes (800–2000 m) are documented for this leguminochorion, higher than for the Southern Afromontane, but lower than for the Drakensberg Alpine Centre (Table 4).

The Northern Highveld Region has some key species in common mostly with the Southern Afromontane, the Drakensberg Alpine Centre and the Summer Rainfall Region (e.g. Rhynchosia totta var. totta and Trifolium africanum var. africanum) (Table 5). The Highveld phytochorion, described by Steenkamp et al. (2005), shows similar, but a more confined pattern extending into the Central Bushveld Region. This leguminochorion is included in the Zambezian Region and Kalahari-Highveld Transition Zone (Goldblatt 1978) and in the Highveld (Steenkamp et al. 2005).

### Drakensberg Alpine Centre (A4)

The areas covered by the Drakensberg Alpine Centre is shown to be in the Mesic Highveld, Drakensberg Grassland and Sub-Escarpment that forms the key bioregions, with Grassland the only biome part of this leguminochorion (Figure 3, Table 2 and 3).

Figure 4 clearly shows that the Drakensberg Alpine Centre falls in a high-rainfall area (mostly >800 mm) with relatively low minimum (<2°C) and maximum (<27°C) temperatures. Owing to the high rainfall, the soil low pH (<6.4) and phosphorus content of <10 mgkg^-1^ is to be expected (Figure 4). Legume species adapted to low soil phosphorus and pH have an important role to play in subtropical and tropical regions (Oberson et al. 2006). This leguminochorion is further defined by a high number of days of heavy frost per year (a maximum of 80 days) and more than six cold spells per year with temperatures lower than -2.5°C on three or more consecutive days (Table 4). Also noteworthy is that this leguminochorion has the highest elevation range of all the leguminochoria (>2000 m).

The Drakensberg Alpine Centre has some mutual key species with the Southern Afromontane and the Northern Highveld Region (e.g. Rhynchosia totta var. totta and Trifolium africanum var. africanum) (Table 5). Lessertia perennans var. perennans has the highest occurrence (diagnostic species) and is not present as key species in any other leguminochoria. No link with the Cape flora can be established when comparing key species. The Afromontane (Goldblatt 1978), Drakensberg Alpine Centre (Van Wyk and Smith 2001), Natal (Linder et al. 2005) and the Drakensberg Alpine (Steenkamp et al. 2005) are included in this leguminochorion.

### Coastal Region (A5)

The Indian Ocean Coastal Belt Bioregion contains most QDGCs found in the Coastal Region, followed by the Lowveld and Sub-Escarpment Savannah Bioregion (Figure 3 and Table 2). In terms of biomes, the Indian Ocean Coastal Belt is highly represented in this leguminochorion, followed by the Savannah biome (Table 3).

High annual rainfall (>800 mm/year), high minimum temperatures (>6°C) and moderate to high maximum temperatures represent the climatic conditions of the Coastal Region (Figure 4). As in the case of most of the “A” leguminochoria, relatively low pH and phosphorus levels as well as non-sodic soils are typical properties of the Coastal Region. The fact that this leguminochorion lies in a frost-free area with extreme maximum temperatures of >40°C (Table 4) could be important when selecting legume species for further evaluation.

The Coastal Region has some key species in common with the Southern Afromontane, the Summer Rainfall and the Northern Mistbelt (e.g. Zornia capensis subsp. capensis, also a diagnostic species) (Table 5). High occurrences of *Chamaecrista mimosoides* and *Eriosema salignum* is also noted. The Tongaland-Pondoland Region has elements of the Afromontane (Goldblatt 1978) and it is confirmed here. This leguminochorion forms part of the Tongaland-Pondoland Region (Goldblatt 1978), the Maputaland-Pondoland Region (Van Wyk and Smith 2001), the Natal and Zambezian Central (Linder et al. 2005) and Core Afromontane and Greater Maputaland (Steenkamp et al. 2005).

### Seasonal Rainfall Group (all-year, winter and summer rainfall) (B)

Regions in South Africa, Lesotho and Swaziland that receive rain throughout the year or in either winter or summer are essentially grouped in this cluster. Cluster “B” is the largest cluster and includes the Generalist Group containing many QDGC with only one legume species. One manifestation of data deficiency encountered in the present study was that many of the grids containing only one legume species were grouped in this “residue” Generalist Group. The Seasonal Rainfall Group is subdivided into the seven leguminochoria: Arid Western Region (B1), Lower-rainfall Cape Floristic Region (B2), Central Arid Region (B3), Generalist Group (B4), Summer Rainfall Region (B5), Northern & Northeastern Savannah Region (B6), Kalahari Bushveld Region (B7).

### Arid Western Region (B1)

The area covered by the Arid Western Region shows that the Namaqualand Hardeveld Bioregion is well represented in this leguminochorion (Figure 3 and Table 2), followed by the Bushmanland Bioregion. The Succulent Karoo and Fynbos are the key biomes present in this leguminochorion (Table 3).

Low annual rainfall (<400 mm) with high minimum and maximum temperatures denotes the Arid Western Region (Figure 4). The high soil pH (>7.5) and medium soil phosphorus content is to be expected in the light of the low rainfall in the region. This is the first leguminochorion noted for its medium (52.4%) and highly sodic soils (14.3%) containing relatively high amounts of sodium (Figure 4). The poor infiltration rate and drainage when the soil is wet and hardness when it is dry are especially problematic for good seed germination and erosion control (Qadir and Oster 2004). The information derived from Schulze (2007) as described in Table 4, shows that the extreme maximum temperatures measured exceed 44°C, with high solar radiation from November to February.

The Arid Western Region has some key species in common with the Lower- and Higher-rainfall Cape Floristic Region (e.g. *Sutherlandia frutescens*), but most key species, mainly belonging to the genus *Aspalathus*, are not common with any other leguminochorion (Table 5). Jürgens (1997) and Goldblatt and Manning (2002) recognised that the Succulent Karoo Region forms part of a greater Cape Flora rather than the Nama-Karoo Region and the present study supports this view. The Succulent Karoo Region, not identified as a phytochorion by Linder et al. (2005), is clearly delineated in this study. The Karoo-Namib Region and Cape Region (Goldblatt 1978), the Namaqualand-Namib Domain and Cape Floristic Region (Cowling et al. 1998), the Gariep Centre, Succulent Karoo and Cape Floristic Region (Van Wyk and Smith 2001), the Namib-Karoo and Cape (Linder et al. 2005) and the Northern Succulent Karoo, Southern Succulent Karoo and the Northwestern Fynbos (Steenkamp et al. 2005) are included in this leguminochorion.

### Lower-rainfall Cape Floristic Region (B2)

**Figure 5.**
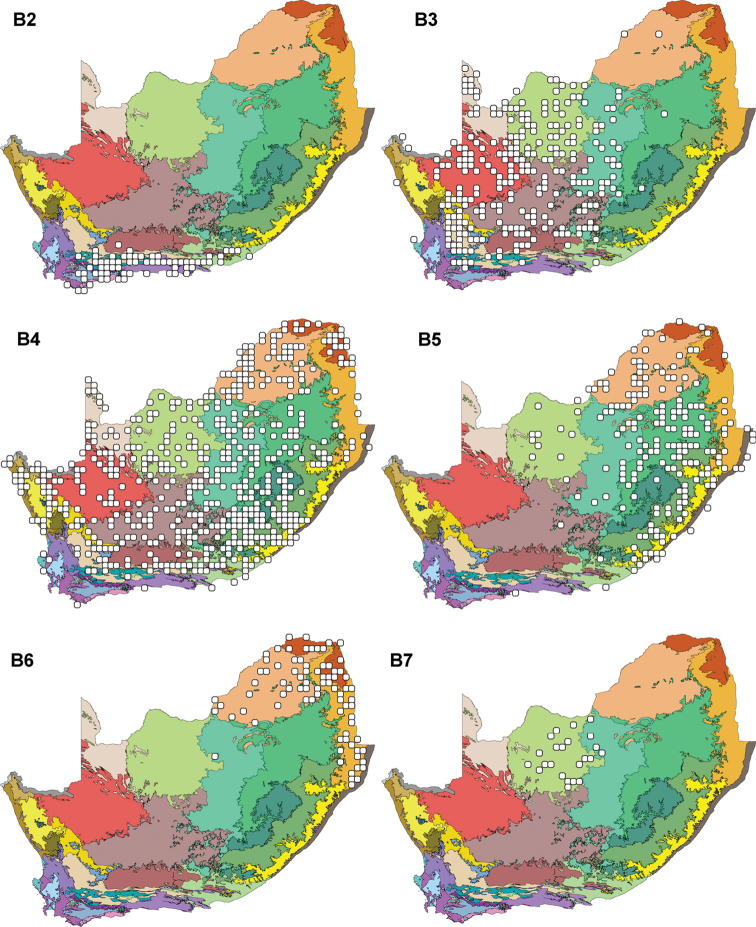
The Leguminochoria **B2–B7** superimposed on the Bioregions of southern Africa. Cluster B (Seasonal Rainfall Group) is divided into the Lower-rainfall Cape Floristic Region (**B2**); the Central Arid Region (**B3**); the Generalist Group (**B4**); the Summer Rainfall Region (**B5**); the Northern & Northeastern Savannah Region (**B6**) and the Kalahari Bushveld Region (**B7**). For the distribution of leguminochorion B1, see Figure 3. The leguminochoria is mapped on bioregions defined by (Rutherford et al. 2006) referring to the legend in Figure 2.

The Albany Thicket and Eastern Fynbos Renosterveld are well represented in the Lower-rainfall Cape Floristic Region (Figure 5 and Table 2). Fynbos is the predominant biome, followed by the Albany Thicket (Table 3).

The annual rainfall figures in Figure 4 indicate that 200–600 mm annual rain is expected for this leguminochorion, but <400 mm is also a probability. Relatively high minimum (2–8°C) and moderate maximum (25–28°C) temperatures are noted. The soil pH varies greatly, with predominantly acidic soils. Half of the soils in this leguminochorion are medium sodic, similar to those of the Arid Western Region (Figure 4). From Table 4 it is clear that this region is mainly semi-arid, cool and dry, with extreme maximum temperatures 36–42°C. It is mainly a frost-free area, but there is a likelihood of <40 days of heavy frost per year.

The majority of key species of the Lower-rainfall Cape Floristic Region are not present in other leguminochoria, indicating their uniqueness to this leguminochorion (Table 5). Some of the key species are mostly in common with the Higher-rainfall Cape Floristic Region, e.g. *Sutherlandia frutescens* and *Aspalathus nigra*. The floristic link of the Cape Region with the Drakensberg Alpine Centre as acknowledged by Goldblatt (1978) and Steenkamp et al. (2005) could not be confirmed with key legume species. A phytochorion termed Southeastern Fynbos, with a similar pattern except for the inclusion of the eastern part of the Cape Region, has also been defined by Steenkamp et al. (2005). The latter authors further speculate that the orientation of the regional mountains could be responsible for the Southeastern (east-west orientation) and Northwestern Fynbos (north-south orientation) phytochoria as described by them. This hypothesis seems supported for the Lower-rainfall Cape Floristic Region, but not for the Higher-rainfall Cape Floristic Region. Goldblatt and Manning’s phytogeographical centres (Goldblatt and Manning 2002) Karoo Mountains, Langeberg, Agulhas Plains and Southeastern Centre closely follow the east-west orientation of the Lower-rainfall Cape Floristic Region. This leguminochorion forms part of the Cape Region (Goldblatt 1978), the Worcester-Robertson Karoo Centre, the Little Karoo Centre and the Cape Floristic Region (Van Wyk and Smith 2001), the Cape (Linder et al. 2005) and the Southeastern Fynbos (Steenkamp et al. 2005).

### Central Arid Region (B3)

The area covered by the Central Arid Region clearly shows that this leguminochorion forms mainly in the dry Eastern Kalahari Bushveld, Bushmanland, Dry Highveld Grassland and Upper Karoo Bioregions (Figure 5 and Table 2). It is noteworthy that the Rainshadow Valley Karoo Bioregion is fairly well represented in this leguminochorion. The Nama-Karoo and Savannah biomes largely represents this leguminochorion (Table 3).

The low annual rainfall of <400 mm noted in Figure 4 is to be expected. The relatively low minimum and high maximum temperatures are also normal for a semi-arid to arid region as Schulze (2007) describes this region in Table 4. The relatively low net primary production as compared to that of the other leguminochoria is noteworthy. The high pH (>7.5) and high soil phosphorus content (>20 mgkg^-1^) defined for the Central Arid Region are expected considering the low annual rainfall (Figure 4). A very small percentage of soils in this leguminochorion are termed medium or highly sodic.

The Central Arid Region lies in the Karoo-Namib Region and the Kalahari-Highveld Transition Zone of Goldblatt (1978). Not surprisingly, most of the key species are also found as key species in the Kalahari Bushveld Region (e.g. Indigofera alternans var. alternans and Indigofera daleoides var. daleoides) (Table 5). Other regions that describe this leguminochorion include the Namib-Karoo and Eastern Karoo (Linder et al. 2005) and the Central Karoo and the Southern Succulent Karoo (Steenkamp et al. 2005).

### Generalist Group (B4)

Bioregions and biomes not present in the Generalist Group are the Fynbos, eastern parts of the Mesic Highveld Grassland, parts of the Sub-Escarpment Grassland and Savannah, Lowveld and Indian Ocean Coastal Belt. The highest percentage bioregions present are the Central Bushveld, Eastern Kalahari Bushveld and Dry Highveld Grassland Bioregions (Figure 5 and Table 2). Savannah and Grassland biomes are most presented (Table 3).

The wide area covered by the Generalist Group is reflected in the wide-ranging climatic and soil conditions shown in Figure 4. Regions with relatively low annual rainfall (<400 mm), low minimum (<2°C) and high maximum (27–35°C) temperatures form mainly part of this leguminochorion. Soils are generally relatively alkaline (pH >7.5) and low in phosphorus (<10 mgkg^-1^). Owing to the wide area covered, Table 4 gives no additional climatic and agrohydrological information.

Notwithstanding its wide distribution, the Generalist Group has various key species that also occur in the Central Arid Region, the Kalahari Bushveld Region and the Albany Centre (e.g. *Melolobium candicans* and *Indigastrum argyraeum*) (Table 5).

### Summer Rainfall Region (B5)

The key bioregions that comprise the Summer Rainfall Region are the Mesic Highveld Grassland and the Central Bushveld, with Grassland and Savannah as key biomes (Figure 5, Table 2 and 3).

The Summer Rainfall Region falls in areas with an annual rainfall of mainly 400–800 mm (Figure 4). Very low minimum temperatures (<4°C) and moderate to high maximum temperatures are recorded. The phosphorus content of soils grouped in the leguminochorion is mainly below 10 mgkg^-1^, with acidic and non-sodic soils (Figure 4). Owing to the wide area covered, Table 4 gives little additional climatic and agrohydrological information.

The Summer Rainfall Region shares some key species with the Southern Afromontane, the Northern Highveld Region and the Central Bushveld Region (e.g. Rhynchosia totta var. totta and *Eriosema salignum*) (Table 5). *Tephrosia semiglabra* is the only key species not present as key species in other leguminochoria. Three of Goldblatt’s phytogeographical regions fall in this leguminochorion, namely the Zambezian Region, the Kalahari-Highveld Transition Zone and the Tongaland-Pondoland Region (Goldblatt 1978).

### Northern & Northeastern Savannah Region (B6)

For the Northern & Northeastern Savannah Region, the Central Bushveld and Lowveld are the two key bioregions, with the Mopane Bioregion listed as a minor component (Figure 5 and Table 2). The Savannah biome represents this leguminochorion in full (Table 3).

Medium annual rainfall (400–800 mm) and relatively high minimum (>6°C) and maximum (27–35°C) temperatures characterise the Northern & Northeastern Savannah Region (Figure 4). Soils are generally acidic, low in phosphorus and non-sodic. This is the only leguminochorion where 16 occurrences of heat waves of >30°C on three or more consecutive days per year are noted in Table 4.

The Northern & Northeastern Savannah Region shares many key species with the Subtropical Lowveld & Mopane Region (e.g. Dichrostachys cinerea subsp. africana var. africana and *Ormocarpum trichocarpum*) (Table 5). Many key species are tree species, e.g. Pterocarpus rotundifolius subsp. rotundifolius and *Faidherbia albida*. This leguminochorion is included in the Zambezian and the Tongaland-Pondoland Regions (Goldblatt 1978), the Zambezian-central (Linder et al. 2005) and the Greater Maputaland (Steenkamp et al. 2005).

### Kalahari Bushveld Region (B7)

It is evident that the Eastern Kalahari Bushveld Bioregion nearly uniquely represents the Kalahari Bushveld Region (Figure 5 and Table 2). In terms of biomes, this leguminochorion lies nearly fully in the Savannah (Table 3).

A relatively medium annual rainfall of 400–800 mm to very low rainfall of <400 mm occurs in the Kalahari Bushveld Region (Figure 4). Low minimum temperatures (<2°C) and high maximum (>27°C) temperatures prevail in this leguminochorion. The slightly acidic (pH = 6.5–7.4), relatively low phosphorus content (<10 mgkg^-1^) and non-sodic soils are described as the main soil properties. Information derived from Schulze (2007) as described in Table 4 indicates that this is a semi-arid, dry area with plains and pans.

The Kalahari Bushveld Region has various key species that are associated with the Central Arid Region and with the Central Bushveld Region (e.g. Indigofera daleoides var. daleoides and also a diagnostic species) (Table 5). *Tephrosia burchellii* has a high occurrence and not found as key species in other leguminochoria. Even though the two leguminochoria are from different bioregions, both lie within the Savannah biome and a floristic link is therefore to be expected. The Kalahari-Highveld Transition Zone (Goldblatt 1978), the Griqualand West Centre (Van Wyk and Smith 2001), the Eastern Karoo and the Karoo Transition (Linder et al. 2005) and the Central Karoo (Steenkamp et al. 2005) form part of this leguminochorion.

### Higher-rainfall Cape Floristic Region (C)

**Figure 6.**
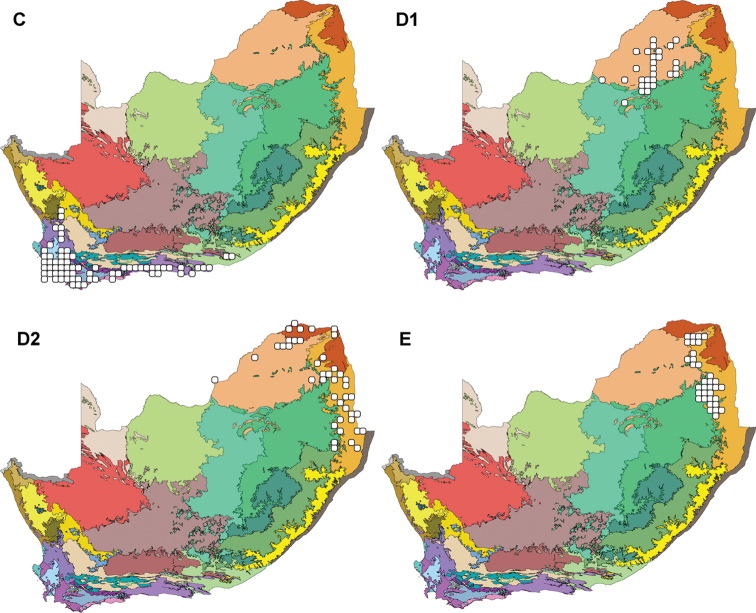
The Leguminochoria **C–E** superimposed on the Bioregions of southern Africa. The Higher-rainfall Cape Floristic Region (Cluster C) and Cluster D (Savannah Group) is divided into the Central Bushveld Region (**D1**) and the Subtropical Lowveld & Mopane Region (**D2**) as well as the Northern Mistbelt (Cluster E). The leguminochoria is mapped on bioregions defined by (Rutherford et al. 2006) referring to the legend in Figure 2.

The key bioregion present in the Higher-rainfall Cape Floristic Region is the Eastern Fynbos Renosterveld with the Southwest Fynbos second highest (Figure 6 and Table 2). This leguminochorion lies entirely in the Fynbos biome (Table 3).

Figure 4 indicates that the annual rainfall is mostly 200–600 mm per year, but that regions of higher rainfall are also included in this leguminochorion. If this is compared with the Lower-rainfall Cape Floristic Region, it is evident that these leguminochoria could be defined individually on the basis of lower and higher annual rainfall. Information derived from Schulze (2007) further confirms the higher rainfall levels in this leguminochorion compared to the Lower-rainfall Cape Floristic Region (Table 4). The minimum temperatures of 2–8°C and maximum temperatures of 25–29°C could be expected in this region. Mostly acidic soils with a wide range of soil phosphorus content is present in this leguminochorion (Figure 4). A high percentage of soils are medium sodic (ESP 6–15%), indicating poor infiltration and drainage, with resultant loss of soil (Qadir and Oster 2004). The leguminochorion forms in a frost-free area (Table 4).

Key species of the Higher-rainfall Cape Floristic Region are found mostly in the Lower-rainfall Cape Floristic Region and only a few in the Arid Western Region (e.g. *Sutherlandia frutescens* and *Indigofera heterophylla*) (Table 5). Most of the key species are not associated with any other leguminochorion, signifying their unique association with this leguminochorion (e.g. *Aspalathus ciliaris* and Aspalathus hispida subsp. hispida). Key species in this region have no floristic link with the Drakensberg Alpine Centre as acknowledged by Goldblatt (1978) and Steenkamp et al. (2005). Goldblatt and Manning’s (2002) phytogeographical centres termed the Northwestern Centre and especially the Southwestern Centre follow the north-south orientation found mainly in this leguminochorion. This leguminochorion forms part of the Cape Region (Goldblatt 1978), the Cape Floristic Region (Van Wyk and Smith 2001), and the Cape (Linder et al. 2005) and the Northwestern and Southeastern Fynbos (Steenkamp et al. 2005).

### Savannah Group (D)

The Savannah Group is subdivided into the Central Bushveld Region (D1) and the Subtropical Lowveld & Mopane Region (D2). Relatively high extreme maximum temperatures with early summer to midsummer rain higher than 400 mm rain is described for this leguminochorion. The region is dry and hot, with a relatively average net primary production (Table 4).

### Central Bushveld Region (D1)

Figure 6 shows that the area covered by the Central Bushveld Region is uniquely formed in the Central Bushveld Bioregion and the Savannah biome (Table 2 and 3), but a number of QDGCs lie in the transitional zone between the Central Bushveld and the Mesic Highveld Grassland Bioregion.

The Central Bushveld Region lies in a zone of annual rainfall of 400–800 mm, with relatively high minimum (2–8°C) and maximum (27–35°C) temperatures (Figure 4). Moderately acidic to neutral soils with low phosphorus levels (<10 mgkg^-1^) as well as non-sodic soils occur in this region (Figure 4). Information derived from Schulze (2007) describes this area as dry and hot or cool (Table 4).

Key species of the Central Bushveld Region are found in the Summer Rainfall Region, the Kalahari Bushveld Region and the Subtropical Lowveld & Mopane Region (*Acacia karroo* and Mundulea sericea subsp. sericea) therefore largely in the Savannah biome (Table 5). *Burkea africana* has a high occurrence and is not noted as key species in other leguminochoria. The Zambezian Region (Goldblatt 1978), the Soutpansberg and Wolkberg Centres (Van Wyk and Smith 2001), the Zambezian-central (Linder et al. 2005) and the Highveld (Steenkamp et al. 2005) form part of this leguminochorion.

### Subtropical Lowveld & Mopane Region (D2)

The Subtropical Lowveld & Mopane Region forms part of the Lowveld, followed by the Central Bushveld and Mopane Bioregions (Figure 6 and Table 2). The Savannah is the only biome that represents the leguminochorion (Table 3).

The expected annual rainfall for the leguminochorion is 400–800 mm per year, but lower and higher rainfall figures are also likely (Figure 4). Relatively high minimum (>6°C) and maximum (27–35°C) temperatures predominate this region. The pH range in the Subtropical Lowveld & Mopane Region varies widely, with soils acidic to alkaline, but mostly below 7.4. Most soils are low in phosphorus, but a considerable portion contains more than 10 mgkg^-1^. Only non-sodic soils are found in this leguminochorion. The main differences between the “D” leguminochoria are that wider ranges of rainfall and soil pH are noted for the Subtropical Lowveld & Mopane Region compared to the Central Bushveld Region. Table 4 shows that extreme maximum temperatures of >40°C are expected in this region.

The key species of the Subtropical Lowveld & Mopane Region are linked mostly with the Northern & Northeastern Savannah Region (e.g. Dichrostachys cinerea subsp. africana var. africana and *Ormocarpum trichocarpum*) (Table 5). This leguminochorion is included in the Zambezian Region and Tongaland-Pondoland Region (Goldblatt 1978), the Zambezian-central (Linder et al. 2005) and Greater Maputaland (Steenkamp et al. 2005).

### Northern Mistbelt (E)

The Mesic Highveld Grassland, Lowveld and Central Bushveld are the key bioregions found in the Northern Mistbelt whereas Savannah is the main biome prevailing in this leguminochorion (Table 2 and 3). It is clear from Figure 6 that this leguminochorion lies in the transitional zone between the aforementioned bioregions.

A high annual rainfall of >800 mm, noted for most of the region included in this leguminochorion, is to be expected for the Northern Mistbelt (Figure 4). Moderate minimum temperatures of 2–8°C and maximum temperatures of 25–29°C are described for this leguminochorion. Acidic (pH <6.4), low phosphorus (<10 mgkg^-1^) and non-sodic soils are present in this leguminochorion (Figure 4). According to Table 4, the leguminochorion falls in a frost-free area, with altitudes of 600–2000 m, slightly lower than in the case of the Drakensberg Alpine Centre.

The Northern Mistbelt shares some key species with the Southern Afromontane, the Coastal Region, the Summer Rainfall Region and the Central Bushveld Region (e.g. Zornia capensis subsp. capensis and Vigna vexillata var. vexillata) (Table 5). A high occurrence of key species is evident in the presence of a large number of diagnostic species, clearly more than in any other leguminochoria. Goldblatt (1978) speculated that the typical Afromontane taxa may have originated from neighbouring lowland flora termed the Coastal Region in this study. The Afromontane (Goldblatt 1978), the Zambezian-central (Linder et al. 2005) and Core Afromontane (Steenkamp et al. 2005) are incorporated in this leguminochorion.

### Species richness, range and growth form

Table 6 gives relevant information on the legume species richness for each leguminochorion as well as the lowest and highest number of legumes collected in the QDGCs within each leguminochorion. The smaller leguminochoria, namely the Higher-rainfall Cape Floristic Region, the Savannah Group and the Northern Mistbelt, have very high species richness, whereas the larger Seasonal Rainfall Group, has a below average species richness. This variation is probably due to the presence of the smaller leguminochoria in the higher-rainfall regions (both temperate and subtropical), while most of the Seasonal Rainfall Group are present in the lower-rainfall (arid) regions. Pausas and Austin (2001) confirm that there is a tendency for species richness to increase with increasing availability of water.

**Table 6.** Quarter degree grid cell (QDGC) percentage, species richness and range within each leguminochorion of southern Africa. Species richness = #Species/#QDGC in each leguminochorion; Species range = lowest and highest species count/QDGC.

Leguminochorion	% QDGC	Species richness	Species range	Species range mean
A1: Southern Afromontane	2.3	7.7 ±6.0	10–62	26.5 ±11.8
A2: Albany Centre	1.3	11.9 ±13.0	15–65	36.3 ±15.9
A3: Northern Highveld Region	2.4	6.5 ±7.7	10–49	26.8 ±9.5
A4: Drakensberg Alpine Centre	2.5	7.4 ±9.1	8–60	25.4 ±13.6
A5: Coastal Region	2.4	9.1 ±10.7	26–104	51.4 ±20.5
B1: Arid Western Region	4.6	5.3 ±4.4	4–47	17.2 ±9.3
B2: Lower-rainfall Cape Floristic Region	4.1	7.3 ±7.2	9–74	23.4 ±12.3
B3: Central Arid Region	16.7	3.0 ±3.3	1–31	5.3 ±4.8
B4: Generalist Group	34.4	2.0 ±1.7	1–21	3.6 ±3.0
B5: Summer Rainfall Region	12.2	3.2 ±2.6	1–25	9.1 ±5.4
B6: Northern & Northeastern Savannah Region	5.0	4.6 ±4.1	5–36	18.1 ±6.8
B7: Kalahari Bushveld Region	1.4	5.9 ±6.7	11–36	20.6 ±7.5
C: Higher-rainfall Cape Floristic Region	4.2	11.9 ±15.3	34–174	69.6 ±29.1
D1: Central Bushveld Region	1.7	12.6 ±16.6	29–198	67.3 ±34.3
D2: Subtropical Lowveld & Mopane Region	2.7	9.3 ±10.4	4–76	47.6 ±13.8
E: Northern Mistbelt	2.1	13.5 ±19.2	28–213	83.6 ±37.1
**Mean**	**100.0**	**7.6**	**12–79**	

The species range (Table 6) within the Sourveld and Mixed Veld Group, shows that the highest range is recorded in the Coastal Region also noted for recording the highest rainfall. The higher species range of the Lower-rainfall Cape Floristic Region within the Seasonal Rainfall Group is to be expected considering the well-known species richness of the Cape Floristic Region. It is noteworthy that a difference in species richness and species range is recorded between the Lower- and Higher-rainfall Cape Floristic Region. The Lower-rainfall Cape Floristic Region shows average records while the Higher-rainfall Cape Floristic Region shows above average records. Also noteworthy is the relatively high species range of the Savannah Group compared to that of the Northern & Northeastern Savannah Region, the two leguminochoria having similar areas covered in mainly the Savannah Bioregion.

**Figure 7.**
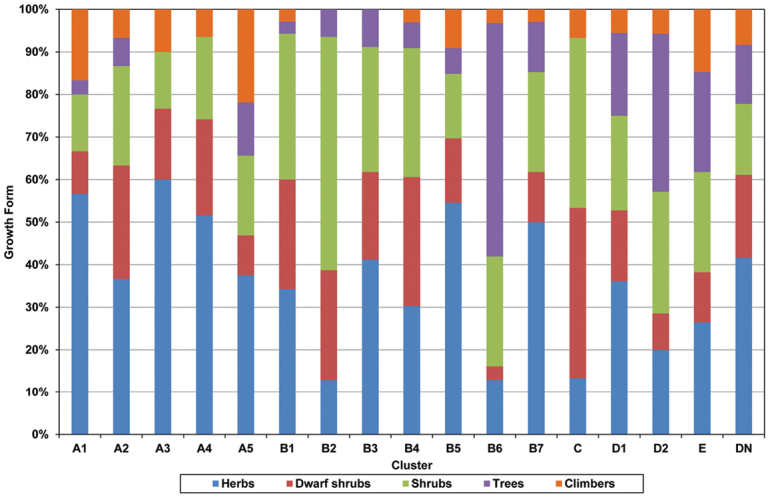
The growth forms of key species recorded in leguminochoria (**A1–E**) of southern Africa. Growth forms are defined as: **1** herb is a small, non-woody seed-bearing plant in which the aerial parts die back at the end of each growing season **2** dwarf shrub is a plant smaller than a shrub which produces wood at its base and has abundant growth branching upward from the base, the upper stems dying back at the end of each growing season **3** shrub is a perennial woody plant less than 10m tall which branches low or near ground level into several main stems although it has no clear trunk **4** tree is a woody plant which grows more than 10m tall, characteristically it has one main stem and **5** climber is a plant with aerial tendrils which it uses to attach itself to a host or surface for support (Germishuizen and Meyer 2003). **DN**: diagnostic species are species with occurrences of 70% or higher. The leguminochoria are termed **A1** Southern Afromontane **A2** Albany Centre **A3** Northern Highveld Region **A4** Drakensberg Alpine Centre **A5** Coastal Region **B1** Arid Western Region **B2** Lower-rainfall Cape Floristic Region **B3** Central Arid Region **B4** Generalist Group **B5** Summer Rainfall Region **B6** Northern & Northeastern Savannah Region **B7** Kalahari Bushveld Region **C** Higher-rainfall Cape Floristic Region **D1** Central Bushveld Region **D2** Subtropical Lowveld & Mopane Region **E** Northern Mistbelt.

The different growth forms of key species for each phytochorion are shown in Figure 7. As highlighted by Pérez-Harguindeguy (2013), growth form may be associated with ecophysiological adaptation, for example where plant species optimise height and foliage arrangement to avoid or resist grazing by certain herbivores, with prostrate growth forms being correlated with high grazing pressure. The dominant growth form in the Sourveld and Mixed Veld Group (A1–A5) is perennial herbs, with a noteworthy number of climber species. Tree species are the least represented of all growth forms. In the Seasonal Rainfall Group (B1–B7), there is a clear increase in the number of shrubs and trees, especially in the Lower-rainfall Cape Floristic Region (i.e. shrubs) and the Northern & Northeastern Savannah Region (i.e. trees). The dominance of dwarf shrubs and shrubs in the Higher-rainfall Cape Floristic Region (C) is similar to the situation in the Lower-rainfall Cape Floristic Region. All growth forms are present in the Savannah Group (D1–D2), with herbs dominating the Central Bushveld Region and trees the Subtropical Lowveld & Mopane Region. Key species of all growth forms in almost equal parts were recorded in the Northern Mistbelt (E). The diagnostic species, i.e. species with occurrences of 70% or higher in a given leguminochoria show dominance in the herb growth form, with nearly equal numbers of the remaining growth forms.

### Legume assemblages

The six assemblages computed by PHYTOTAB-PC are listed in Table 7. Group 1 includes the southern and western Cape Region covering the Succulent Karoo and Fynbos biomes. Group 2 includes two relatively low-rainfall leguminochoria and the Generalist Group covering the Nama Karoo and western Savannah. Group 3 represents the Albany Centre, which is noted as a single entity, indicating no floristic links with any of the other leguminochoria. The inclusion of the north-eastern parts of South Africa into Group 4 that covers the Savannah biome is to be expected. The Drakensberg Alpine Centre in Group 5 has no apparent floristic link with the Afromontane regions and forms part of the Grassland biome. Group 6 is a well-defined Afromontane region that includes the coastal areas below the Drakensberg.

**Table 7.** Classification of Leguminochoria of southern Africa in assemblages.

Assemblages	Leguminochoria included within an assemblage
1	Arid Western Region (B1), Lower-rainfall Cape Floristic Region (B2), Higher-rainfall Cape Floristic Region (C)
2	Central Arid Region (B3), Generalist Group (B4), Kalahari Bushveld Region (B7)
3	Albany Centre (A2)
4	Northern & Northeastern Savannah Region (B6), Central Bushveld Region (D1), Subtropical Lowveld & Mopane Region (D2)
5	Northern Highveld Region (A3), Drakensberg Alpine Centre (A4), Summer Rainfall Region (B5)
6	Southern Afromontane (A1), Coastal Region (A5), Northern Mistbelt (E)

**Figure 8.**
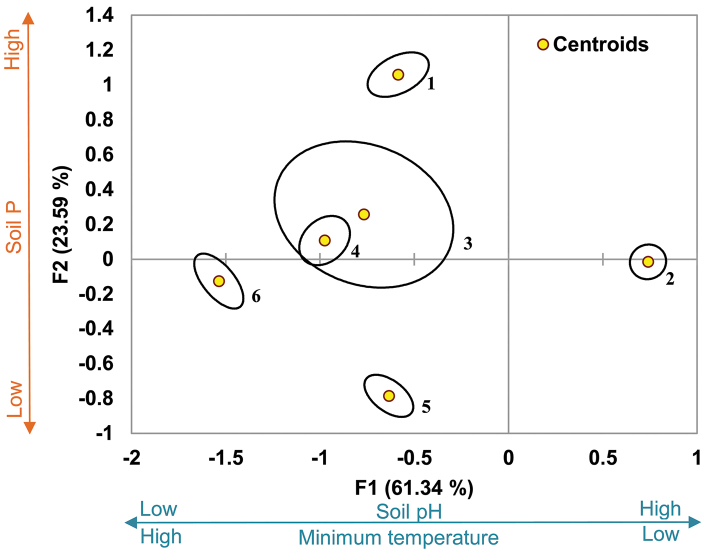
Discriminant analysis for legume assemblages of southern Africa. Only the centroids and not all observations are shown. Confidence ellipses around the centroids and drivers for Factor 1 (soil pH and minimum temperatures) and Factor 2 (soil phosphorus) are shown. The legume assemblages are **1** Arid Western Region, Lower-rainfall Cape Floristic Region, Higher-rainfall Cape Floristic Region **2** Central Arid Region, Generalist Group, Kalahari Bushveld Region **3** Albany Centre **4** Northern & Northeastern Savannah Region, Central Bushveld Region, Subtropical Lowveld & Mopane Region **5** Northern Highveld Region, Drakensberg Alpine Centre, Summer Rainfall Region and **6** Southern Afromontane, Coastal Region, Northern Mistbelt.

The result of the Pearson’s correlation matrix for the legume assemblages grouped by PHYTOTAB-PC is shown in Table 8. The Pearson’s correlation matrix indicates that for F1, soil pH and mean annual minimum temperature (negative) are the main drivers for distinguishing among legume assemblages, whereas for F2, soil phosphorus level is the main driver. The result for the discriminant analysis is shown in Figure 8 where only the centroids and not all observations are shown due to the large dataset (largely overlying groups). The F1 function (soil pH and mean annual minimum temperature) accounts for 61.43% of the independent variables and the F2 function (soil phosphorus content) accounts for 23.59% of the independent variables (Figure 8).

**Table 8.** Pearson’s correlation coefficients for Leguminochoria assemblages of southern Africa.

Variables	F1	F2	F3
Mean annual rainfall (mm)	-0.555	-0.550	0.149
Maximum temperature (°C)	0.545	0.145	**0.695**
Minimum temperature (°C)	-**0.646**^a^	0.683	0.332
Soil phosphorus (mgkg^-1^)	0.391	**0.817**	-0.227
Soil pH (H_2_O)	**0.798**	0.516	0.195

a Values in bold are different from 0 with a significance level alpha = 0.05

Group 6 (Southern Afromontane, Coastal Region and the Northern Mistbelt) positioned to the left on the F1 axis contain species adapted to low soil pH and high minimum temperatures (Figure 8). Group 2 (Central Arid Region, Generalist Group and the Kalahari Bushveld Region) positioned to the right on the F1 axis contain species adapted to high soil pH and low minimum temperatures. Group 1 (Arid Western Region, Lower-rainfall Cape Floristic Region and the Higher-rainfall Cape Floristic Region) positioned at the upper level on the F2 axis contain species adapted to average soil pH and minimum temperatures and high soil phosphorus as opposed to Group 5 (Northern Highveld Region, Drakensberg Alpine Centre and Summer Rainfall Region) that contain species adapted to low soil phosphorus. Group 3 (Albany Centre) and Group 4 (Northern & Northeastern Savannah Region, Central Bushveld Region, Subtropical Lowveld & Mopane Region) are positioned more to the centre and contain species adapted to average soil pH, minimum temperatures and soil phosphorus. It is clear that legume assemblages were grouped mainly based on soil differences, followed by temperature, while rainfall was least important. Other studies, however, showed that the most important abiotic factors that control species distribution are temperature and moisture (Skarpe 1986, Woodward 1987, Ruiz-Vega 1994, Bond et al. 2003). It was corroborated by Greve (2011) that rainfall is the most important variable for the distribution of African vegetation for all vegetation types.

Davis’ report (2011) on climate change in southern Africa indicate that small increases in temperature are unlikely to affect plant distribution in a desert (partly enclosed in the extreme northern part of the Arid Western Region), whereas in an arid to semi-arid ecotone (enclosed in the Arid Western Region, Central Arid Region, Kalahari Bushveld Region and Central Bushveld Region), plants could disappear owing to a higher biophysical vulnerability to climate change. In addition to temperature and moisture, Bond et al. (2003) and Midgley et al. (2007) highlight the significant effect of fire on South African vegetation. Fynbos (enclosed in the Lower-rainfall and Higher-rainfall Cape Floristic Region), at least in the more mesic areas, is a fire-dependent ecosystem and could support a forest or thicket. Summer-rainfall areas with an annual rainfall >650 mm (mainly the Southern Afromontane, Northern Highveld Region, Drakensberg Alpine Centre, Summer Rainfall Region and Northern Mistbelt) could become forest with the exclusion of fire, and with <650 mm could show no compositional change in fire-intolerant forest or thicket species (climate-dependent grassy ecosystems) (mainly the Central Arid Region, Generalist Group, Northern and Northeastern Savannah Region, Kalahari Bushveld Region, Central Bushveld Region and Subtropical Lowveld & Mopane Region).

## Conclusions

The Sourveld and Mixed Veld Group represents a group of legume species found mostly in the Grassland and Eastern Coastal Regions and to a lesser extent in the Albany Thicket and Lowveld Regions. The largest leguminochorion, the Seasonal Rainfall Group, includes all regions except the Higher-rainfall Cape Floristic Region and the Northern Mistbelt, being distinctly formed leguminochoria. The Lower-rainfall Cape Floristic Region shares part of the Eastern Fynbos-Renosterveld Bioregion with the Higher-rainfall Cape Floristic Region, although it is also found in the Albany Thicket. The Savannah Group forms part of the Central Bushveld, Lowveld & Mopane Bioregions, similar to the Northern & Northeastern Savannah Region. The smallest leguminochorion, the Northern Mistbelt, is found in the transitional zone between the Mesic Highveld Grassland, the Lowveld and the Central Bushveld Bioregions.

For the Sourveld and Mixed Veld Group, a commonality is the relatively high annual rainfall figures, low pH (< 6.4) and non-sodic soils noted. The minimum and maximum temperatures differ widely within the “A” clusters. It is clear that the Southern Afromontane can be distinguished from the Northern Highveld Region purely based on rainfall figures. The colder conditions that prevail in the Drakensberg Alpine Centre compared to those in the Southern Afromontane are evident from the climatic data, a conclusion also reached by Steenkamp et al. (2005). The Seasonal Rainfall Group shows that the annual rainfall is relatively low and that a relatively high maximum temperature prevails. The soil phosphorus content and pH of this cluster vary widely, but some soils are medium to highly sodic. The difference in climate between the two Cape Floristic Regions is evident where the Lower-rainfall Cape Floristic Region includes areas with annual rainfall figures of <400 mm, while the Higher-rainfall Cape Floristic Region includes areas with annual rainfall figures of >400 mm. The medium annual rainfall and high minimum and maximum temperatures are distinct attributes of the Savannah Group. The climatic and soil conditions for the Northern & Northeastern Savannah Region and the Savannah Group are without doubt comparable owing to similar areas covered. The Northern Mistbelt has a relatively high annual rainfall figure and moderate temperatures, similar to those of the Sourveld and Mixed Veld Group. A low soil phosphorus and pH value are recorded for the Northern Mistbelt.

The six legume assemblages that were identified are geographically sound. The separation of the Albany Centre is unexpected and merits further investigation, especially since some key species were noted as common to other leguminochoria and in the light of Van Wyk and Smith’s (2001) observation that floristic elements of many other regions converge in this centre.

It is concluded in this first time study on the African continent that a single plant family, in this case the Leguminosae, do not necessarily follow vegetation units. The vegetation units can be correlated with limiting environmental factors even on a national scale using rainfall, soil pH, soil phosphorus and temperature. In this study, members of the Leguminosae formed clusters based on:

1) Distinctive patterns reflecting either vegetational or geographical regions, for example the Arid Western Region, the Lower- and Higher-rainfall Cape Floristic Region, the Albany Centre and the Central Bushveld Region; 2) Non-distinctive vegetational patterns, for example the Generalist Group where most vegetational types are present or where residue grids (mainly those with fewer than three species) were grouped; 3) Functional types, for example the Northern Highveld Region with largely herbs and Northern & Northeastern Savannah Region largely trees are the main growth form.

With the exception of a few indigenous legume species (e.g. *Lablab purpureus*, *Lotononis bainesii* and *Vigna unguiculata*) successfully integrated in present-day pasture systems, the vast untapped genetic resources available for pasture screening or soil conservation programs, are evident from this study.

## Supplementary materials

Supplementary material 1 Statistical results of the clustering analysis using the Agglomerative Hierarchical Clustering method. Data type: phylogenetic data This dataset is made available under the Open Database License (http://opendatacommons.org/licenses/odbl/1.0/). The Open Database License (ODbL) is a license agreement intended to allow users to freely share, modify, and use this Dataset while maintaining this same freedom for others, provided that the original source and author(s) are credited.Marike Trytsman, Robert H. Westfall, Philippus J. J. Breytenbach, Frikkie J. Calitz, Abraham E. van Wyk

Supplementary material 2 Species recorded in each leguminochorion (A1–E) of southern Africa. Data type: species data This dataset is made available under the Open Database License (http://opendatacommons.org/licenses/odbl/1.0/). The Open Database License (ODbL) is a license agreement intended to allow users to freely share, modify, and use this Dataset while maintaining this same freedom for others, provided that the original source and author(s) are credited.Marike Trytsman, Robert H. Westfall, Philippus J. J. Breytenbach, Frikkie J. Calitz, Abraham E. van Wyk

Supplementary material 3 The predominant mean annual rainfall and minimum and maximum temperatures expressed as a percentage for southern African leguminochoria. Data type: meteorological data This dataset is made available under the Open Database License (http://opendatacommons.org/licenses/odbl/1.0/). The Open Database License (ODbL) is a license agreement intended to allow users to freely share, modify, and use this Dataset while maintaining this same freedom for others, provided that the original source and author(s) are credited.Marike Trytsman, Robert H. Westfall, Philippus J. J. Breytenbach, Frikkie J. Calitz, Abraham E. van Wyk

Supplementary material 4 The predominant soil phosphorus content, pH level and exchangeable sodium percentage (ESP) expressed as a percentage for southern African leguminochoria. Data type: meteorological data This dataset is made available under the Open Database License (http://opendatacommons.org/licenses/odbl/1.0/). The Open Database License (ODbL) is a license agreement intended to allow users to freely share, modify, and use this Dataset while maintaining this same freedom for others, provided that the original source and author(s) are credited.Marike Trytsman, Robert H. Westfall, Philippus J. J. Breytenbach, Frikkie J. Calitz, Abraham E. van Wyk
