# Supplementary material for: Diversity and biogeographical patterns of legumes (Leguminosae) indigenous to southern Africa
Source: PhytoKeys. 2016 Oct 4;(70):53–96. doi: 10.3897/phytokeys.70.9147 (PMC5088704; doi:10.3897/phytokeys.70.9147)
Supplement: Supplementary material 1 — Statistical results of the clustering analysis using the Agglomerative Hierarchical Clustering method. [file phytokeys-070-053-s001.pdf]

Supplementary file 1. Statistical results of the clustering analysis using the Agglomerative Hierarchical Clustering method.

A. Variance decomposition for the optimal classification using XLSTAT 2010.6.01 Software (Addinsoft):

	**Absolute**	**%**
Within-class	14.521	88.8
Between-classes	1.833	11.2
Total	16.354	100.0

B. Results by class:

			**Cluster**		
	**A**	**B**	**C**	**D**	**E**
Within-class variance	28.698	7.713	58.008	43.081	53.756
Minimum distance to centroid	3.177	0.975	5.596	4.762	5.780
Average distance to centroid	5.208	2.474	7.463	6.433	7.150
Maximum distance to centroid	9.233	8.523	11.251	11.748	10.890
Centroid QDGC	2928AB	2727CD	3418BA	2529AC	2431CC
