# Supplementary material for: Diversity and biogeographical patterns of legumes (Leguminosae) indigenous to southern Africa
Source: PhytoKeys. 2016 Oct 4;(70):53–96. doi: 10.3897/phytokeys.70.9147 (PMC5088704; doi:10.3897/phytokeys.70.9147)
Supplement: Supplementary material 3 — The predominant mean annual rainfall and minimum and maximum temperatures expressed as a percentage for southern African leguminochoria. [file phytokeys-070-053-s003.pdf]

Supplementary file 3. The predominant mean annual rainfall and minimum and maximum temperatures expressed as a percentage for southern African leguminochoria.

	**Rainfall (mm)**					**Minimum temperature (°C)**				**Maximum temperature (°C)**			
Cluster	**<400**	**200–600**	**400–800**	**600–1000**	**>800**	**<2**	**0–4**	**2–8**	**>6**	**<27**	**25–29**	**27–35**	**>33**
A1				**56.8**	43.2		**46.0**	**46.0**	8.0	13.5	**70.3**	16.2	
A2		9.5	**66.7**	19.1	4.7		28.6	**47.6**	23.8		**95.2**	4.8	
A3			**84.6**	15.4		33.3	**46.2**	20.5		15.4	**48.7**	35.9	
A4		2.5	17.5	22.5	**57.5**	**70.0**	30.0			**80.0**	17.5	2.5	
A5			18.0	7.6	**74.4**			20.5	**79.5**		**59.0**	41.0	
B1	**86.7**[a]	10.7	2.6			1.3		41.3	**57.4**	1.3	36.0	**48.0**	14.7
B2	31.8	**43.9**	19.7	3.0	1.6		1.5	**80.3**	18.2	12.1	**56.1**	31.8	
B3	**76.0**	8.1	15.5	0.4		**49.1**	26.2	22.9	1.8		7.0	**63.1**	29.9
B4	**42.6**	9.5	35.6	7.5	4.8	**40.8**	16.7	30.3	12.2	8.3	25.1	**53.9**	12.7
B5	7.1	3.5	**50.3**	21.3	17.8	**31.0**	24.4	28.9	15.7	17.8	**40.6**	38.6	3.0
B6	6.2	18.5	**59.3**	11.1	4.9	1.2	6.2	42.0	**50.6**		18.5	**80.3**	1.2
B7	39.1	13.1	**47.8**			**91.3**	8.7					**69.6**	30.4
C	4.4	**41.2**	29.4	16.2	8.8		16.2	**60.3**	23.5	14.7	**80.9**	4.4	
D1			**100.0**			3.5	17.9	**78.6**			39.3	**60.7**	
D2	14.0	16.3	**46.5**	14.0	9.2			48.8	**51.2**		20.9	**76.8**	2.3
E		3.0	23.5	23.5	**50.0**		8.8	**76.5**	14.7	11.8	**70.6**	17.6	

[a]Bold-formatted figures indicate the highest percentage rainfall and temperatures in a cluster.

A1: Southern Afromontane; A2: Albany Centre; A3: Northern Highveld Region; A4: Drakensberg Alpine Centre; A5: Coastal Region; B1: Arid Western Region; B2: Lower-rainfall Cape Floristic Region; B3: Central Arid Region; B4: Generalist Group; B5: Summer Rainfall Region; B6: Northern & Northeastern Savannah Region; B7: Kalahari Bushveld Region; C: Higher-rainfall Cape Floristic Region; D1: Central Bushveld Region; D2: Subtropical Lowveld & Mopane Region; E: Northern Mistbelt.
