# Supplementary material for: Diversity and biogeographical patterns of legumes (Leguminosae) indigenous to southern Africa
Source: PhytoKeys. 2016 Oct 4;(70):53–96. doi: 10.3897/phytokeys.70.9147 (PMC5088704; doi:10.3897/phytokeys.70.9147)
Supplement: Supplementary material 4 — The predominant soil phosphorus content, pH level and exchangeable sodium percentage (ESP) expressed as a percentage for southern African leguminochoria. [file phytokeys-070-053-s004.pdf]

Supplementary file 4. The predominant soil phosphorus content, pH level and exchangeable sodium percentage (ESP) expressed as a percentage for southern African leguminochoria.

	**Phosphorus ($mgkg^{-1}$)**				**pH (H_2O)**				**ESP**		
Cluster	**<10**	**5–35**	**>20**	**<6.4**	**5.5–7.4**	**6.5–7.4**	**6.5–8.4**	**>7.5**	**1-6**	**6-15**	**>15**
A1	**94.6**[a]	5.4		**81.1**	18.9				**100.0**		
A2	23.8	**76.2**		**33.3**	23.8	14.3	28.6		**100.0**		
A3	**100.0**			**82.1**	12.8		5.1		**100.0**		
A4	**90.0**	10.0		**87.5**	12.5				**100.0**		
A5	**79.5**	20.5		**66.7**	30.8	2.5			**100.0**		
B1	1.3	**57.3**	41.4	2.7	13.3	14.7	33.3	**36.0**	33.3	**52.4**	14.3
B2	4.6	**78.8**	16.6	21.2	**28.8**	7.6	25.8	16.6	**50.0**	**50.0**	
B3	32.1	32.8	**35.1**	2.7	8.8	18.8	16.2	**53.5**	**88.4**	6.2	5.4
B4	**47.4**	31.2	21.4	15.3	20.3	17.6	19.2	**27.6**	**90.7**	6.2	3.1
B5	**77.7**	22.3		**56.9**	22.8	14.7	4.1	1.5	**99.2**	0.8	
B6	**55.6**	44.4		18.5	**40.7**	18.5	22.3		**100.0**		
B7	**100.0**				21.7	**47.8**	17.4	13.1	**100.0**		
C	4.4	**92.7**	2.9	**50.0**	32.4	7.4	8.8	1.4	38.5	**61.5**	
D1	**85.7**	14.3		**60.7**	39.3				**100.0**		
D2	**60.5**	39.5		**27.9**	**27.9**	20.9	18.6	4.7	**100.0**		
E	**67.7**	32.3		**79.4**	17.7		2.9		**100.0**		

[a]Bold-formatted figures indicate the highest percentage soil P, pH and ESP in a cluster.

A1: Southern Afromontane; A2: Albany Centre; A3: Northern Highveld Region; A4: Drakensberg Alpine Centre; A5: Coastal Region; B1: Arid Western Region; B2: Lower-rainfall Cape Floristic Region; B3: Central Arid Region; B4: Generalist Group; B5: Summer Rainfall Region; B6: Northern & Northeastern Savannah Region; B7: Kalahari Bushveld Region; C: Higher-rainfall Cape Floristic Region; D1: Central Bushveld Region; D2: Subtropical Lowveld & Mopane Region; E: Northern Mistbelt.
